# Correlations for Concentration Polarization and Pressure Drop in Spacer-Filled RO Membrane Modules Based on CFD Simulations

**DOI:** 10.3390/membranes11050338

**Published:** 2021-05-01

**Authors:** Boram Gu, Claire S. Adjiman, Xiao Yun Xu

**Affiliations:** 1 Department of Chemical Engineering, Imperial College London, London SW7 2AZ, UK; c.adjiman@imperial.ac.uk; 2 School of Chemical Engineering, Chonnam National University, 77 Yongbong-ro, Buk-gu, Gwangju 61186, Korea

**Keywords:** reverse osmosis (RO), feed spacers, computational fluid dynamics (CFD), concentration polarization (CP), pressure drop, correlations

## Abstract

Empirical correlations for mass transfer coefficient and friction factor are often used in process models for reverse osmosis (RO) membrane systems. These usually involve four dimensionless groups, namely Reynolds number (Re), Sherwood number (Sh), friction factor (*f*), and Schmidt number (Sc), with the associated coefficients and exponents being obtained by fitting to experimental data. However, the range of geometric and operating conditions covered by the experiments is often limited. In this study, new dimensionless correlations for concentration polarization (CP) modulus and friction factor are presented, which are obtained by dimensional analysis and using simulation data from computational fluid dynamics (CFD). Two-dimensional CFD simulations are performed on three configurations of spacer-filled channels with 76 combinations of operating and geometric conditions for each configuration, covering a broad range of conditions encountered in RO membrane systems. Results obtained with the new correlations are compared with those from existing correlations in the literature. There is good consistency in the predicted CP with mean discrepancies less than 6%, but larger discrepancies for pressure gradient are found among the various friction factor correlations. Furthermore, the new correlations are implemented in a process model with six spiral wound modules in series and the predicted recovery, pressure drop, and specific energy consumption are compared with a reference case obtained by ROSA (Reverse Osmosis System Analysis, The Dow Chemical Company). Differences in predicted recovery and pressure drop are up to 5% and 83%, respectively, highlighting the need for careful selection of correlations when using predictive models in process design. Compared to existing mass transfer correlations, a distinct advantage of our correlations for CP modulus is that they can be directly used to estimate the impact of permeate flux on CP at a membrane surface without having to resort to the film theory.

## 1. Introduction

Spiral wound modules are the most widely used membrane modules in a reverse osmosis (RO) process owing to a number of desirable features, such as their higher packing density than plate-and-frame modules and lower fouling propensity than hollow fiber modules [1]. As multiple membrane sheets are rolled around a central tube in a spiral wound module, spacers are inserted to keep membrane sheets separate. The presence of spacers affects the performance and energy consumption of an RO process, due to their influences on mass and momentum transfer. Concentration polarization (CP) is mitigated by enhanced mixing induced by spacers while pressure drop is increased due to raised frictional resistance. In order to predict membrane performance and pumping energy requirements, it is essential to gain a quantitative understanding of the mass and momentum transfer processes in a membrane module. This has motivated numerous experimental and computational studies on the effects of spacers on CP and pressure drop [2,3,4,5,6,7,8,9,10,11,12,13,14,15,16,17,18,19,20,21,22,23,24,25,26,27,28,29,30].

Considerable progress has been made on developing correlations for mass transfer coefficient and pressure drop in a membrane channel, with a number of empirical correlations available in the literature [3,5,6,7,8,10,11,12,13,26,27,31,32,33]. Mass transfer correlations are commonly used for quantitative description of CP at a membrane surface by using the film theory [2,34,35]. More recently, computational fluid dynamics (CFD) simulations have been performed to investigate the effects of spacers on mass transfer coefficient and pressure drop [17,18,19,20,21,22,23,24,25,26,27,28,29], and furthermore, to establish mathematical correlations incorporating geometric features of spacers [26,27]. However, the range of geometric and operating conditions covered by existing correlations is limited. Therefore, it is important to evaluate the performance of existing correlations and to modify these to suit for a wide range of RO conditions. An ideal correlation should be derived in the presence of membrane permeation, incorporate geometric information of spacers, and be valid for a wide range of RO operating conditions.

Correlations for mass transfer coefficient and pressure drop typically involve the following dimensionless groups: Sherwood number (*Sh*), Reynolds (*Re*), and Schmidt (*Sc*) number for mass transfer coefficient and friction factor (*f*) and Reynolds (*Re*) for pressure drop. It should be noted that different definitions of *f* and *Sh* exist due to different characteristic lengths used. Geometric parameters representing spacer filaments are taken into account either in the dimensionless groups as a characteristic length or as an additional term.

A number of empirical correlations were derived from mass transfer experiments in an electrodialysis process using an electrochemical cell without a membrane [5,7,8,27]. As a result, these correlations do not directly account for permeation velocity and should be coupled with the film theory and 1D convection-diffusion model to correlate permeation velocity and CP. Schock and Miquel [30] introduced a mathematical expression for the hydraulic diameter of a spacer-filled channel so that the dimensionless variables (i.e., *Re* and *Sh*) contain geometric features of spacers. Their experiments were performed on an RO membrane module, making their correlation well suited for prediction of CP and pressure drop in an RO process. The number of spacers tested in their work, however, is limited to five types. Da Costa et al. [11,12,13] carried out a series of experimental studies on ultrafiltration (UF) membranes and spacers. By examining a variety of spacer geometries, they developed a single correlation for *Sh* that incorporates a number of geometric characteristics of spacers [13]. Nevertheless, their correlation was derived at velocity conditions more appropriate for a UF rather than an RO process [26]. More recently, Koutsou et al. [26,27] presented correlations for *Sh* and pressure drop in 3D spacer geometries based on CFD simulations and experimental measurements in an electrochemical cell with prototype spacers. Although various spacer geometries were considered under typical operating conditions for RO processes, the correlations were derived for each spacer geometry separately, without inclusion of geometric parameters.

The present study focuses on deriving dimensionless correlations for CP modulus and friction factor for a wide range of geometric and operating conditions relevant to a spacer-filled channel of an RO membrane module. This is undertaken by means of CFD simulations of two-dimensional (2D) models of spacers with varying geometry and operating conditions. By means of dimensional analysis, a correlation for CP modulus is formulated instead of a mass transfer coefficient (or *Sh*), so that CP can be calculated directly without having to couple the film theory with a process model. In order to simulate a spacer-filled feed channel in an RO process as realistically as possible, the CFD model accounts for the effect of permeation through RO membranes by coupling the solution–diffusion model with the governing equations for fluid flow and solute transport. It is expected that the derived correlations are able to capture mass and momentum transfer phenomena in the presence of membrane and feed spacer for a wide range of RO operating conditions.

In the following section, the CFD modelling approach is described, which is followed by the derivation of dimensionless correlations. Next, the key performance indicators obtained from CFD simulations are presented, together with the obtained correlations for CP modulus and friction factor. Derived correlations are also compared with existing correlations in the literature. Finally, the derived and existing correlations are implemented in a process model with multiple spiral wound modules, where both feed and permeate flows are accounted for in a real size module. In doing so, the impact of using different correlations on predicted module performance, such as recovery, pressure drop, and specific energy consumption in an RO process, can be assessed.

## 2. Methods

Details of the CFD models are described in this section including model geometry, governing equations, and boundary conditions. This is followed by the derivation of correlations for CP and pressure drop in spacer-filled channels.

### 2.1. CFD Simulations

**Figure 1:**
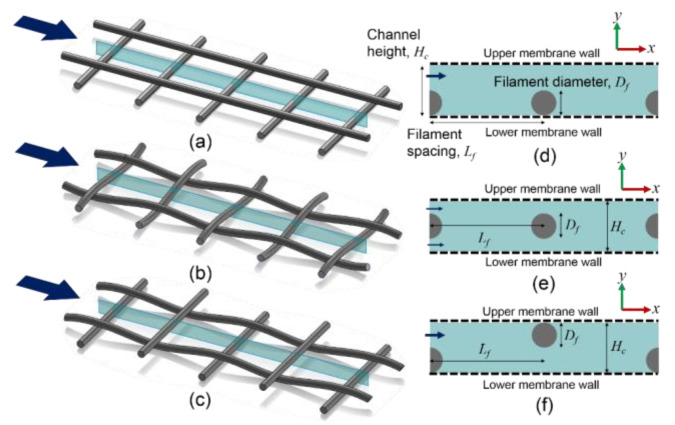
Three-dimensional spacer geometries (**a**–**c**) and their cross-sections along the main flow path (**d**–**f**). (**a**) Nonwoven spacer, (**b**) fully woven spacer, (**c**) partially woven spacer. 2D cross-sections: (**d**) cavity, (**e**) submerged, and (**f**) zigzag configuration.

Two-dimensional (2D) models of different types of spacers are created by taking the cross-sections of spacer filaments in a feed channel, which represents the main flow path. Figure 1 illustrates three spacer types: nonwoven, partially woven and fully woven spacers [36,37]. When transverse filaments are normal to the inflow direction and are also at 90º with the axial filaments, three types of cross-sections can be created: cavity, submerged, and zigzag configurations as shown in Figure 1 [18,20,21,22,24,25,38]. In this study, 2D models of the three cross-sectional configurations are constructed as simplified representations of spacer-filled channels with upper and lower permeable membrane walls. Since each configuration has a repeating pattern of filament arrangement along the flow path, a unit cell is built to simulate a spacer-filled channel. For the cavity and submerged configurations, a single elemental unit consisting of two filaments can present the repeating pattern in a channel, while the zigzag configuration requires two elemental units consisting of three filaments. For the sake of consistency in geometry, two elemental units are included in each model, which consists of one circular filament in the middle and two half filaments at the entrance and exit of each spacer-filled channel.

In the fluid domain, governing equations for steady-state laminar flow and solute transport are solved for an incompressible and Newtonian fluid. The continuity, Navier–Stokes and convection-diffusion equations are given in Equations (1) to (3), respectively.

(1) ∇·u=0

(2) $$\rho u\cdot \nabla u=-\nabla p+\mu \nabla^{2}u$$

(3) $$\nabla \cdot \left(D_{sw}\nabla u\right)-\nabla \cdot \left(uc\right)=0$$

where **u** is the fluid velocity vector, *ρ* the density of the fluid, *μ* the viscosity of the fluid, *p* the pressure, *c* the solute concentration and *D_sw_* the diffusivity of solute in water. A dilute aqueous solution with a single solute is assumed and the fluid properties are approximated by those of pure water.

A fully developed velocity profile is imposed at the inlet, while a constant pressure is specified at the outlet. Spacer filaments are treated as no-slip walls, where zero velocities are prescribed. For solute transport, a flat concentration profile is applied at the inlet, and a no flux condition is imposed at the outlet. At membrane walls, the solution–diffusion model is applied, which allows a realistic description of the transport phenomena through RO membranes [39,40,41]. By coupling with the van’t Hoff equation to calculate osmotic pressure, water, and solute fluxes in the solution–diffusion model can be expressed as:

(4) $$J_{w}=A[(p-p_{p})-\kappa (c-c_{p})]$$

(5) $$J_{s}=B\left(c-c_{p}\right)$$

where *J_w_* is the water flux, *J_s_* the solute flux, *A* the hydraulic conductivity, *B* the solute permeability, *p_p_* the permeate pressure, *c_p_* the permeate concentration, and *κ* the osmotic factor, which is assumed constant under an isothermal condition. 

By introducing two assumptions: (i) permeate pressure is constant (here, *p_p_* = 0) and (ii) permeate concentration is determined by the solute to water flux ratio (*c_p_* = *J_s_*/*J_w_*), water and solute fluxes can be expressed as a function of feed concentration and pressure [37].

(6) $$J_{w}=\frac{A\left(p-\kappa c\right)-B+\sqrt{{\left\{A\left(p-\kappa c\right)+B\right\}}^{2}+4\kappa ABc}}{2}$$

(7) $$J_{s}=B\left[\frac{A\left(p+\kappa c\right)+B-\sqrt{{\left\{A\left(p-\kappa c\right)+B\right\}}^{2}+4\kappa ABc}}{2\kappa A}\right]$$

Equations (6) and (7) are used to provide boundary conditions at the upper and lower membrane walls, where the following conditions are applied:

(8) $$u_{l,up}=\left[\begin{array}{cc} 0 & J_{w} \end{array}\right]$$

(9) $$u_{l,low}=\left[\begin{array}{cc} 0 & -J_{w} \end{array}\right]$$

(10) $$-n\cdot \left(D_{sw}\nabla c-uc\right)=-J_{s}$$

where **n** is the normal vector and **u_l,up_** and **u_l,low_** are the leaking velocities at the upper and lower membrane walls, respectively.

Model geometries are created for 45 combinations of filament diameter *D_f_* and filament spacing *L_f_* (details are provided later). The fluid domains are discretized into quadrilateral elements of varying sizes with denser mesh near the upper and lower membrane walls in order to ensure adequate resolution in the mass transfer boundary layer. Local mesh refinement is also performed in regions where circular filaments are in contact with membrane walls in the cavity and zigzag configurations. The number of mesh elements for the baseline cases (*D_f_* = 0.5 mm and *L_f_* = 4.5 mm) are 14,400, 16,200, and 9000 for the cavity, submerged and zigzag configurations, respectively. Mesh independence study is performed by increasing the mesh density until changes in averaged water and solute fluxes are less than 3 × 10^−5^ %. Moreover, the local concentration in the vicinity of center filament and membrane wall, at *x* = *L_f_* + 0.1 mm and *y* = 0.01 mm for the cavity and submerged configuration and *y* = *H_c_* – 0.01 mm for the zigzag configuration, is monitored and changes between the coarsest and finest meshes are below 0.02% for three configurations.

CFD simulations are carried out using COMSOL Multiphysics 5.1, which is based on the finite element method. Linear formulation is chosen for both velocity and pressure and the concentration field. The stationary solutions are obtained by the nonlinear solver using the fully coupled and automatic damped Newton method. The MUltifrontal Massively Parallel Spacer direct solver (MUMPS) is used to solve the system of linear algebraic equations with a convergence criterion of 10^−6^ for the relative error in the solution.

### 2.2. Selection of Geometric and Operating Conditions

**Figure 2:**
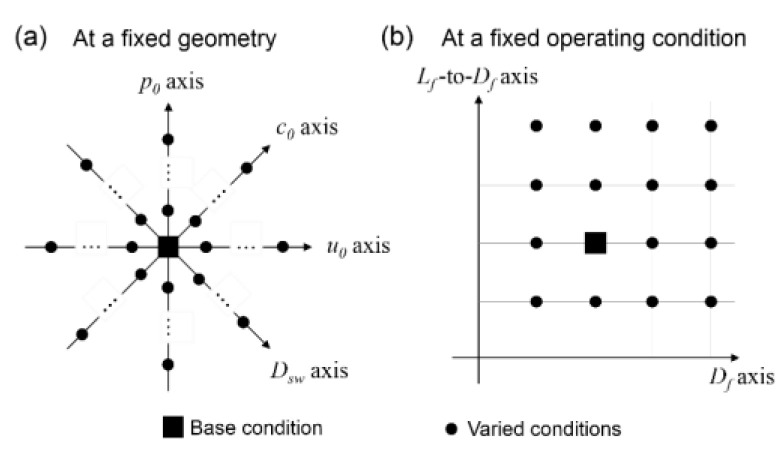
Schematic illustration of strategy for varying operating and geometric conditions. (**a**) at a fixed geometry (**b**) at a fixed operating condition.

Six operating and geometric parameters are varied in CFD simulations: average inlet velocity *u_0_*, solute diffusivity *D_sw_*, inlet concentration *c_0_* and outlet pressure *p_0_*, filament spacing *L_f_*, and filament diameter *D_f_*. Each operating parameter is varied one at a time while the other parameters are kept the same as in the corresponding baseline case. As shown in Figure 2, geometric variables *L_f_* and *D_f_* are simultaneously varied with the operating variables being fixed. For each configuration, a total of 76 cases are simulated as listed in Table 1. Spacers’ geometric parameters *L_f_* and *D_f_* are varied by selecting different feed channel height *H_c_* and *L_f_* to *H_c_* ratio, with *D_f_* and *H_c_* being related as: *D_f_* = 0.5*H_c_*. The baseline inlet concentration 603.45 mol/m^3^ is equivalent to 35 kg/m^3^ of an aqueous sodium chloride solution, which can approximate seawater. The hydraulic conductivity *A* and solute permeability *B* are selected within reported ranges [37,42,43,44]. The osmotic factor *κ* is obtained at temperature of 298.15 K.

### 2.3. Dimensional Analysis and Derivation of Correlations

Dimensional analysis using the Buckingham *Pi* theorem [45] is performed to identify the dimensionless groups involved in the correlations for CP modulus and pressure drop. A detailed procedure to formulate the correlations can also be found in Section A in the Supporting Information. 

#### 2.3.1. Correlation for CP Modulus

There are eight parameters involved in determining CP: CP modulus *M_CP_*, fluid density *ρ*, dynamic viscosity *μ*, solute diffusivity in fluid *D_sw_*, cross-velocity *u_c_*, transmembrane velocity *u_t_* (or water flux at membrane walls *J_w_*), filament diameter *D_f_*, and filament spacing *L_f_*. In this study, a slightly different definition of CP modulus is used to facilitate its implementation in an RO process model.

(11) $$M_{CP}=\frac{c_{m}-c_{p}}{c_{0}-c_{p}}$$

where *c_m_* is the feed concentration at membrane walls and *c_0_* the inlet concentration. According to the Buckingham *Pi* theorem, the above dimensional parameters can be grouped into five independent dimensionless groups (8 − 3 = 5). By choosing *ρ*, *μ*, *D_f_* as repeating variables, five dimensionless groups are formed, including *M_CP_* and 4 dimensionless groups commonly used in momentum and mass transfer, such as Re and Sc. The dimensionless groups *Re_c_*, *Re_t_*, *Sc*, and *GR* are defined as:

(12) $$Re_{c}=\frac{\rho u_{c}D_{f}}{\mu }$$

(13) $$Re_{t}=\frac{\rho u_{t}D_{f}}{\mu }\;\;\mathrm{or}\;\;\frac{\rho J_{w}D_{f}}{\mu }$$

(14) $$Sc=\frac{\mu }{\rho D_{sw}}$$

(15) $$GR=\frac{L_{f}}{D_{f}}$$

Here the filament diameter *D_f_* is used as a characteristic length in *Re_c_* and *Re_t_*. *GR*, the filament spacing to diameter ratio, allows the inclusion of an important geometric feature of a spacer-filled channel. Their functional relationship can be expressed as follows:

(16) $$M_{CP}-1=CRe_{c}{}^{\alpha }{\left(m\cdot Re_{t}\right)}^{\beta }Sc^{\gamma }GR^{\delta }$$

where *C*, *α*, *β*, *γ,* and *δ* are coefficient and exponents that are dependent on the spacer configuration (cavity, submerged, and zigzag). Although *Re_t_* could be considered negligible compared to *Re_c_* due to transmembrane velocity being usually several order of magnitude smaller than cross-velocity, the term is kept in Equation (16) for the time being with an adjustment factor *m*. This is discussed later in Section 3.1.1 in relation with CFD results for the effects of transmembrane velocity on CP moduli. 

#### 2.3.2. Correlation for Pressure Drop

Pressure drop in a spacer-filled channel is influenced by the following parameters: pressure gradient *dp*/*dx*, *u_c_*, *ρ*, *μ*, *D_f_*, and *L_f_*. The transmembrane velocity *u_t_* is not included as its effect on axial pressure drop is negligible due to its small magnitude compared to the cross-velocity. Analysis using the Buckingham *Pi* theorem yields three dimensionless groups, which can be related as follows:

(17) $$f=C^{\prime }Re_{c}{}^{\alpha^{\prime }}GR^{\delta^{\prime }}+\zeta$$

with

(18) $$f=\frac{D_{f}}{\rho u_{c}{}^{2}}\frac{dp}{dx}$$

where *f* is the derived friction factor, *C*′, *α*′, *δ*′, and *ζ* are coefficients and exponents to be determined by experimental or numerical simulation data. The local pressure gradient *dp*/*dx* in Equation (18) can be replaced by Δ*p*/(2*L_f_*), which is the pressure drop per unit length in the fluid domain. 

#### 2.3.3. Parameter Estimation

The coefficients and exponents in Equations (16) and (17) are estimated by data fitting, using results obtained from 2D CFD simulations. For each case, the simulation results for *M_CP_*, *u_c_*, *u_t_*, and Δ*p* are averaged over the entire fluid domain, and spatially averaged values are used for data fitting. A wide range of values for the dimensionless parameters are considered by varying operating and geometric conditions (*Sc* = 111–4475, *Re_c_* = 1.12–274, *Re_t_* = 7.82 × 10^−4^–6.70 × 10^−3^, and *GR* = 4–12). Since transmembrane velocity, *u_t_*, is a key parameter that determines CP, a range of *u_t_* (3–12 × 10^−6^ m/s) are obtained by varying feed concentration and pressure in the simulations. The coefficient and exponents in Equation (16) are estimated by solving the following:

(19) $$\min\sum_{n=1}^{N=76}{\left[M_{CP,n}{}^{CFD}-M_{CP,n}\left(C,\alpha ,\beta ,\gamma ,\delta \right)\right]}^{2}$$

where *M_CP_^CFD^* is the CP modulus obtained from CFD simulations, *M_CP_* (*C*, *α*, *β*, *γ*, *δ*) the CP modulus calculated by Equation (16) with a set of parameters, *n* the index of data and *N* the total number of data. To find the values of *C*, *α*, *β*, *γ*, and δ that minimize the sum of the squared errors of CP moduli, the built-in simplex algorithm in MATLAB 2015b is utilized. Since there might exist multiple local minima, it is critical to provide a good initial guess, which is obtained by differentiating Equation (16) together with the corresponding CFD data. 

### 2.4. Implementation of Correlations in a Process Model

The new correlations obtained in this study and existing correlations available in the literature are implemented in a predictive model for a spiral wound module [42], where feed and permeate streams are independently accounted for in a real size module, and the predicted performances using different correlations are compared. The correlations are implemented as follows:

- Correlations for CP (obtained in this study) are coupled with the solution–diffusion model described in Equations (4) and (5).
- Existing correlations for mass transfer coefficient are implemented using the film theory.
- All these are incorporated in a spiral wound module model, where mass balances for the feed and permeate streams are performed separately.

Among the existing correlations in the literature, choices are made from those derived under the conditions similar to RO processes. Two correlations for mass transfer coefficient are chosen: Schock and Miquel [30] and Koutsou et al. [27] for Sherwood number, which are expressed in Equations (20) and (21), respectively.

(20) $$Sh=0.065Re_{c}^{0.875}Sc^{0.25}$$

(21) $$Sh=0.2Re_{c}^{0.57}Sc^{0.40}$$

Definition of *Sc* is the same as given in Equation (14), but *Sh* and *Re_c_* have different characteristic lengths; *d_h_* and *D_f_* for Equations (20) and (21), respectively. The derived expression for *d_h_* of spacer-filled channels in [30] is expressed as:

(22) $$d_{h}=\frac{4\varepsilon }{2/H_{c}+(1-\varepsilon )4/L_{f}}$$

where *ε* is the voidage of the spacer-filled channel. The selected correlations for pressure drop are those of Schock and Miquel [30] and Koutsou et al. [26], as given in Equations (23) and (24), respectively.

(23) $$\frac{dp}{dx}=f_{SM}\frac{\rho u_{c}{}^{2}}{2d_{h}}\mathrm{where}\;f_{SM}=6.23Re_{c}^{-0.3}\;\;$$

(24) $$\frac{dp}{dx}=f_{K}\frac{Re_{c}^{2}\rho \nu^{2}}{D_{f}{}^{3}}\;\;\mathrm{where}\;f_{K}=2.3Re_{c}^{-0.31}\;\mathrm{at}\;\frac{L_{f}}{D_{f}}=6\;\;\mathrm{and}\;f_{K}=0.8Re_{c}^{-0.19}\;\;\mathrm{at}\;\frac{L_{f}}{D_{f}}=8$$

where *f_SM_* and *f_K_* are the friction factors in the respective correlations, and *ν* the kinematic viscosity. Two correlations, as in Equation (24), were derived using 3D CFD simulations with a constant *D_f_* (=1 mm). The main reason for selecting the aforementioned correlations is that they were derived under RO operating conditions, which usually yield a feed flow velocity below 0.4 m/s [23]. Two additional correlations for mass transfer coefficient [30,42,46,47] and pressure drop [42,48] are also included in the comparison of module performance, which are frequently used in membrane process models when spacer specifications are not available.

(25) $$Sh=1.85Re_{c}{}^{1/3}Sc^{1/3}{\left(\frac{d_{h,empty}}{L}\right)}^{1/3}$$

(26) $$\frac{dp}{dx}=\frac{12k_{sp}\mu }{{\left(0.5H_{c}\right)}^{2}}u_{c}$$

where *k_sp_* represents wall roughness in a channel due to spacer filaments. The negative sign for pressure drop correlations in Equations (18), (23), (24) and (26) is omitted in order to make friction factors positive, but it is added when *dp*/*dx* is implemented in a process model. Equation (25) is for a flat channel without spacers, in which the hydraulic diameter, *d_h_*_,*empty*_, correspond to that of an empty channel. 

In the model for a spiral wound module, mass balance equations are expressed in the form of differential equations so that spatial variations along the feed and permeate flow directions can be taken into account [42,43,44]. For process simulations a typical arrangement is considered, which has six spiral wound modules arrayed in series in a pressure vessel [49,50,51], and only one pressure vessel is included. The number of leaves and membrane width are estimated using the model presented in [42] based on the given module diameter in the product data sheet [52]. Permeability constants (*A* and *B* in Equations (4) and (5)) and frictional coefficients (*k_sp_* in Equation (26)) are also estimated by comparing predicted recovery, feed pressure drop and permeate concentration with those from ROSA (Reverse Osmosis System Analysis, The Dow Chemical Company), the latter serve as reference data for comparison purposes. The estimated permeabilities and friction coefficients values are listed in Table 2, as well as module and process specifications used to simulate RO processes. 

## 3. Results and Discussions

### 3.1. Analysis of CFD Results

In this section, CFD results are presented and analyzed with a focus on predicted performance indicators, including water flux, CP modulus, and pressure drop. Velocity vectors and concentration profiles obtained from the CFD simulations are included in Section B in the Supporting Information. 

#### 3.1.1. Water Flux and CP Modulus

**Figure 3:**
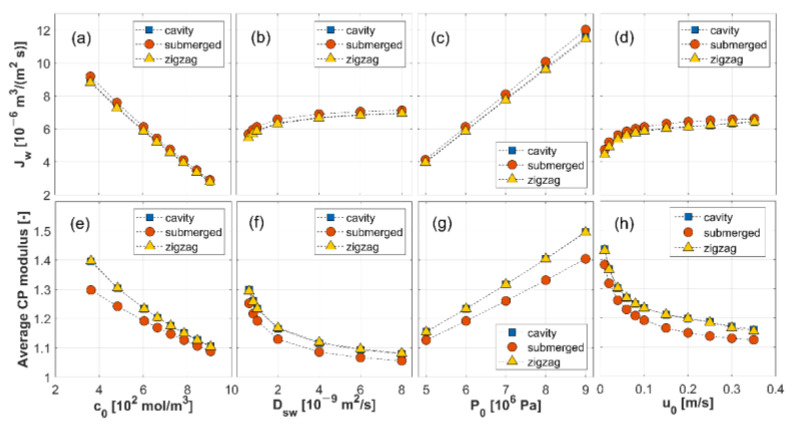
Average water fluxes (top) and CP moduli (bottom) in the cavity, submerged and zigzag configurations for varying inlet concentration *c*_0_ (**a**,**e**), diffusivity of solute in water *D_sw_* (**b**,**f**), outlet pressure *p*_0_ (**c**,**g**), and inlet velocity *u*_0_ (**d**,**h**).

Average water fluxes and CP moduli in three configurations under various operating conditions (the baseline condition and conditions 1–30) are shown in Figure 3. It is clear that feed concentration and pressure have a direct impact on water flux-water flux increases with a decrease in feed concentration or an increase in feed pressure. This is because water permeation is driven by the net pressure defined as (Δ*p* – *κ*Δ*c*), according to the solution–diffusion model in Equation (4). As can be seen in Figure 3a,c,e and g, there appears to be a direct relationship between average water flux and CP modulus when only feed concentration or pressure is varied, suggesting that CP is strongly influenced by transmembrane velocity. This finding justifies the inclusion of *Re_t_* in the CP modulus correlation in Equation (16); if *Re_t_* was neglected, the effects of transmembrane velocity caused by different feed concentrations and pressures on CP moduli would not be captured. On the other hand, increasing solute diffusivity and inlet flow velocity causes water flux to rise until it plateaus, as shown in Figure 3b,d. The increased water flux could be due to mitigated CP by accelerated diffusion of solutes back to the bulk flow at high solute diffusivity and by enhanced mixing near membrane walls at high flow velocity. This is supported by Figure 3f,h, where CP moduli are reduced as solute diffusivity or inlet flow velocity increases. The trend of water flux plateauing at high inlet velocity in Figure 3d also indicates that there is a limit to the impact of enhanced mixing.

Comparisons among the different configurations suggest that the submerged configuration has a slightly higher water flux within the ranges studied. CP moduli are lower in the submerged configuration than in the cavity and zigzag configurations, due to the absence of direct contact between spacer filaments and membrane walls. The maximum difference between the cavity and submerged configurations are 7.8% for water flux and 7.0% for CP modulus. The response of CP modulus to increasing inlet velocity appears to be biphasic, with exponential decays for inlet velocity up to 0.1 m/s and almost linear reduction thereafter for the cavity and zigzag configurations. This behavior is not observed in the submerged configuration. It is possible that the direct contact between filaments and membrane walls in the cavity and zigzag configurations is responsible for the biphasic behavior, which is influenced by the extent of flow recirculation and stagnation.

**Figure 4:**
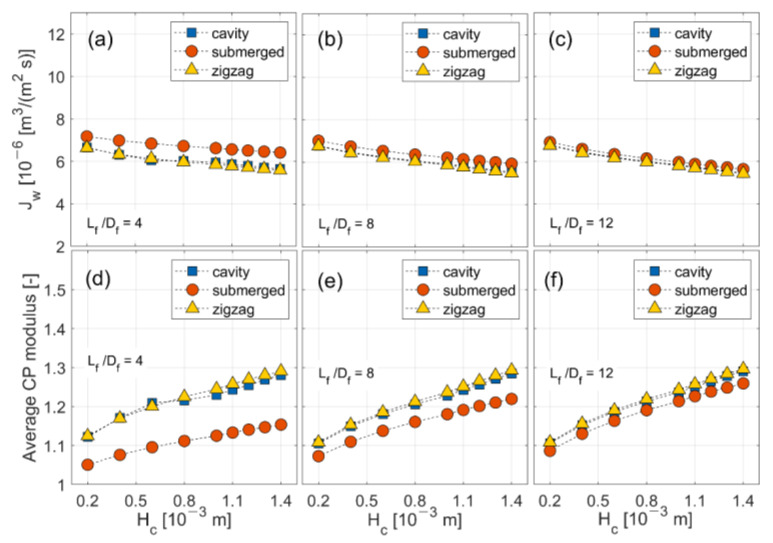
Average water fluxes (top) and CP moduli (bottom) in the cavity, submerged and zigzag configurations for varying channel height at selected filament length to diameter (*L_f_*/*D_f_*) ratios. (**a**,**d**): *L_f_*/*D_f_* =4; (**b**,**e**): *L_f_*/*D_f_* =8; (**c**,**f**): *L_f_*/*D_f_* =12.

Average water fluxes and CP moduli are displayed with respect to channel height at different filament lengths to diameter (*L_f_*/*D_f_*) ratios in Figure 4. The effect of channel height is obvious: water flux is reduced and CP modulus is elevated with an increase in channel height. Compared to the cavity configuration, the submerged configuration offers 2.1–13.5% higher water flux and 1.8–9.9% lower CP modulus by varying the geometric conditions. The results also indicate that denser spacer mesh (i.e., at small *L_f_*/*D_f_* ratios) achieves a slightly better performance.

Similar biphasic behavior is observed in the simulation results with varying geometric conditions, most notably at small *L_f_*/*D_f_* ratio in Figure 4d, where there is a logarithmic increase in CP modulus up to a channel height of around 0.8 mm and an almost linear increase thereafter for the cavity and zigzag configurations. Combined with the observation described previously in relation to Figure 3h, it appears that in the cavity and zigzag configurations CP moduli behave differently in two distinguished regimes, which can be separated using a critical Reynolds number, Re_crit_, based on the cross-flow velocity and characteristic length, *D_f_* (= 0.5*H_f_*). Therefore, a critical Reynolds number is introduced in this study to more accurately correlate CP moduli with operating and geometric conditions for the cavity and zigzag configurations.

#### 3.1.2. Pressure Drop and Friction Factor

**Figure 5:**
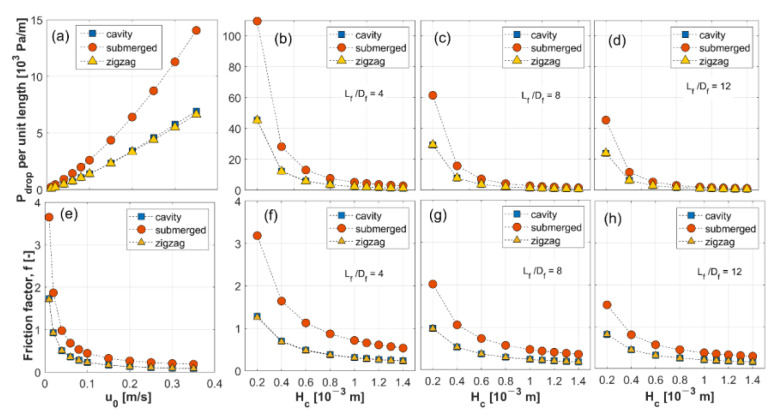
Pressure drop per unit length (top) and friction factor (bottom) in the cavity, submerged and zigzag configurations with respect to inlet velocity (**a**,**e**) and channel height with *L_f_*/*D_f_* =4 (**b**,**f**), *L_f_*/*D_f_* =/8 (**c**,**g**), *L_f_*/*D_f_* =12 (**d**,**h**).

Pressure drop per unit length is calculated using the predicted axial pressure drop from CFD and the channel length, while the corresponding friction factors are calculated by using Equation (18). Results for varying inlet velocities and geometric conditions are presented in Figure 5. As expected, pressure drop increases as inlet velocity increases. Regardless of the operating and geometric conditions, the submerged configuration appears to have the highest pressure drop among the three configurations. This is because the spacer filaments are located in the middle of the channel, which forces the flow to split into two streams when passing around the filament, causing increased local velocity. On the other hand, little variation in pressure drop is found with varying inlet concentration, solute diffusivity and outlet pressure, justifying the formulation of the correlation for pressure drop, as expressed in Equation (17).

The effects of geometric variables on pressure drop can also be deduced from the results for different *L_f_*/*D_f_* ratios (Figure 5b–d). As the channel height increases, pressure drop per unit length decreases rapidly. At the same channel height, higher *L_f_*/*D_f_* ratios result in less pronounced pressure drops per unit length. Since *L_f_*/*D_f_* ratio affects the void volume in a channel, it has a strong influence on the mean velocity and pressure drop. Friction factors show a similar trend to pressure drop per unit length except for varying inlet velocities. The definition of friction factor in Equation (18) dictates that it is inversely proportional to the square of cross-flow velocity, which results in the trend shown in Figure 5e.

### 3.2. Correlations for CP Modulus and Friction Factor

Values for the parameters in the CP modulus (Equation (16)) and friction factor (Equation (17)) correlations are obtained by using the CFD simulation results, and these are shown in Table 3, together with the corresponding absolute percentage errors (APE) from data fitting. The adjustment factor m in Equation (16) is 10^4^ in order to make the exponents of *Re_t_* and *Re_c_* be of a similar order of magnitude. Both CP modulus (*M_CP_*) and friction factor (f) correlations have a good fit to the CFD data with a mean APE less than 1.5% for all configurations. There are two sets of parameters for the *M_CP_* correlation for the cavity and zigzag configurations, depending on the critical Reynolds number, *Re_crit_*, which is found to be 24.4–31.0 for varying inlet velocities (Figure 3h) and geometric conditions (Figure 4d). As a result, *Re_crit_* is assumed to be 25.5 for both the cavity and zigzag configurations.

For all configurations, values for the exponents of *Re_c_* and *Re_t_* (i.e., *α* and *β*) reveal that CP modulus is more sensitive to the transmembrane velocity than cross-flow velocity. The value for *β* indicates that the relationship between *Re_t_* and *M_CP_* is almost linear, whereas the value for *α* suggests that *Re_c_* has a stronger influence on *M_CP_* at low *Re_c_* than at high *Re_c_*. It is also noted that the cavity and zigzag configurations have similar parameter values for the given regime, but there are differences between the two regimes. The value for γ, exponent of Sc, is assumed to be constant regardless of the flow regimes but to vary with the filament configuration. For the cavity and zigzag configurations, there is only a slight difference in *α* and *β* between the two regimes. However, values for *δ* are markedly different, leading to different effects: in the low *Re_c_* regime, *M_CP_* decreases slightly with an increase in *L_f_*/*D_f_* ratio, as displayed in Figure 4d–f. In the high *Re_c_* regime, *δ* tends to zero for both configurations, suggesting a negligible effect of *L_f_*/*D_f_* on *M_CP_*. For the submerged configuration, *δ* is very different from that for the other configurations; the positive sign of *δ* means that *M_CP_* increases with an increase in *L_f_*/*D_f_*, as shown in Figure 4d–f. The coefficient *C* varies with hydrodynamics and geometry, hence giving different values depending on configurations and flow regimes.

Estimated parameter values for the friction factor correlations are comparable for the cavity and zigzag configurations. The value for *α′*, the exponent of *Re_c_*, implies an inverse relation between *f* and *Re_c_* as can be seen in Figure 5e. For the exponent of *L_f_*/*D_f_* ratio, *δ′*, its values capture the fact that f decreases more rapidly with an increase in *L_f_*/*D_f_* in the submerged configuration than in the cavity and zigzag configurations, as displayed in Figure 5f–h. The parameters *C′* and *ζ* for the submerged configuration are found to be approximately 3.4–4.5 times larger than those for the cavity and zigzag configurations. This suggests the submerged configuration has a larger friction factor in most situations. Further comparisons between the CFD results and derived correlations can be found in Section C in the Supporting Information. 

### 3.3. Comparison with Existing Correlations

Before being implemented in a process model, the new correlations are compared with exiting correlations described in Section 2.4. Comparisons are made for a range of flow velocities (0.095–0.35 m/s) using spacer specifications listed in Table 2. The chosen velocity range corresponds to the range of hydraulic Reynolds numbers examined by Schock and Miquel [30]. Koutsou et al. derived the mass transfer correlation in Equation (21) from their experimental results on prototype spacers with a similar *L_f_*/*D_f_* ratio to commercial spacers (*L_f_*/*D_f_* = 8 and 12 and mesh angle = 90°) [26,27]. However, the filament diameter (1 mm) adopted in their work far exceeded that of commercial spacers (0.27–0.385 mm) based on the study by Schock and Miquel on four feed spacers in spiral wound RO modules [30].

#### 3.3.1. CP Modulus and Water Flux

Results for CP modulus and the corresponding water flux obtained using our new correlations are compared with those of Schock and Miquel [30] and Koutsou et al [26,27]. Since the latter are based on Sherwood number (or mass transfer coefficient), it is necessary to involve water flux to relate mass transfer coefficient to CP modulus [30]. In addition, it should be borne in mind that Koutsou et al. [27] measured the mass transfer coefficient in an electrochemical cell in the absence of membrane.

**Figure 6:**
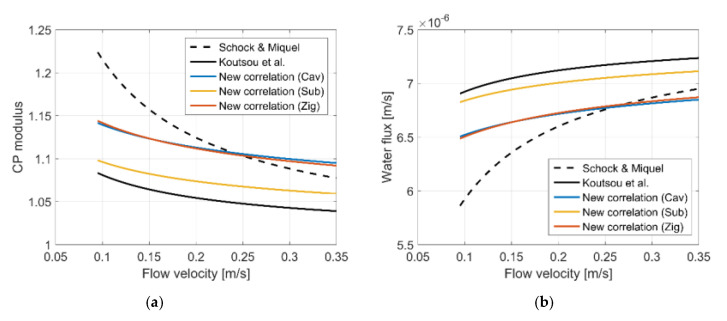
Comparisons of CP modulus and water flux calculated by different correlations: Sherwood number correlations of Schock and Miquel [30] and Koutsou et al. [27] expressed in Equations (20) and (21), respectively, and our new correlations. (**a**) CP modulus and (**b**) water flux.

Figure 6 shows that the predicted behavior of CP modulus with respect to varying flow velocity is similar between our new correlations and those of Koutsou et al. with discrepancies of less than 6%, which corresponds to approximately 6% difference in water flux. On the other hand, CP moduli and water fluxes obtained with the correlation by Schock and Miquel are more sensitive to changes in flow velocity in that the CP modulus declines more rapidly with increasing flow velocity, leading to a more pronounced increase in water flux. In addition, the Schock and Miquel correlation tends to predict higher CP modulus and consequently lower water flux than others, particularly in the low velocity range. Similar findings were also reported by Koutsou et al. [27], and they attributed this discrepancy to uncertainties related to the indirect determination of mass transfer coefficient (or Sherwood number) using the solution–diffusion model and film theory. However, it should also be noted that these correlations were derived for different ranges of geometric parameters.

Results from our new correlations for the three configurations lie between those of Schock and Miquel and Koutsou et al., as can be seen in Figure 6a, although our correlations for the cavity and zigzag configurations predict a slightly higher CP modulus when flow velocities exceed 0.25 m/s. Mean and maximum differences compared to the correlations of Schock and Miquel and Koutsou et al. are summarized in Table 4. It is difficult to ascertain which correlation is more accurate for RO applications. This will be discussed further in Section 3.5 in relation to limitations of the CFD model.

#### 3.3.2. Pressure Gradient

**Figure 7:**
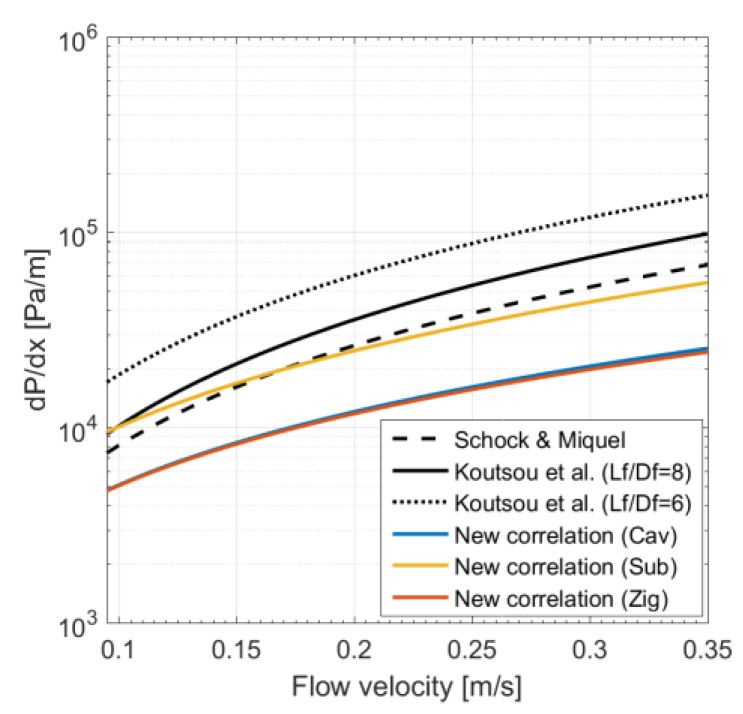
Comparison of pressure gradient calculated by different correlations: Schock and Miquel [30] and Koutsout et al. [26] expressed in Equations (23) and (24), respectively, and our new correlations for three spacer configurations.

Values for pressure gradient *dp*/*dx* obtained with our correlations are compared with those of Schock and Miquel and Koutsou et al. in Equations (23) and (24). As shown in Figure 7, the correlations of Koutsou et al. for *L_f_*/*D_f_* = 6 and *L_f_*/*D_f_* = 8 predict larger pressure drops than that of Schock and Miquel and our new correlations. For the particular spacer simulated here (Table 2), *L_f_*/*D_f_* = 7.3, which lies between the *L_f_*/*D_f_* ratios covered by the correlations of Koutsou et al.; the latter appear to over-predict *dp*/*dx* by 2.3 times compared to the Schock and Miquel correlation for the range of flow velocities examined. This is consistent with the findings reported in Koutsou et al. [26]. The mean and maximum percentage differences between the new and existing correlations are summarized in Table 5.

The submerged correlation is closer to that of Schock and Miquel compared to the correlations for the cavity and zigzag configurations. Although Koutsou et al. also derived their correlations based on CFD results, there are a number of differences between their work and the present study: (i) their CFD models are 3D; (ii) a periodic condition with a constant mean-pressure gradient between inlet and outlet faces is imposed with the assumption of impermeable membrane walls; and (iii) Koutsou et al. simulated only four data points at flow velocities of 0.02, 0.08, 0.1, and 0.15 m/s, with a fixed channel height of 2 mm, whereas our simulations cover a wider range of operating and geometric conditions.

### 3.4. Simulation Results from Process Model

Correlations for CP and friction factor are implemented in a process model, and nine simulations are performed as listed in Table 6, under three operating conditions that lead to a water recovery of approximately 50%. Case 1 corresponds to the reference case, which is set to reproduce the simulation results obtained with ROSA. Cases 2 to 5 are designed to test the influence of different friction factor correlations while using the same CP correlation as in the reference case, whereas Cases 6 to 9 allow the influence of CP correlations to be determined when the same pressure drop correlation (Equation (26)) is used. Comparisons are made for the predicted water recovery, specific energy consumption, and pressure drop along six modules. Results for all simulation cases are compared with those of Case 1, and the relative differences are reported in Table 6. 

Table 6 shows that changing the friction factor correlations (Cases 1 to 5) has an almost negligible effect on the predicted recovery, with differences of up to 1.5% at the highest feed flowrate, although the predicted pressure drop varies considerably. Nevertheless, the magnitude of pressure drop is too small to make a notable effect on the predicted specific energy consumption, which has a relative difference of less than 2% for all conditions examined. It is worth noting that the largest differences are observed in Case 5 where the correlation of Koutsou et al. for *L_f_*/*D_f_* = 8 is used. Since this correlation was derived over a narrow flow velocity range (up to 0.15 m/s), it is not surprising that larger differences are found at higher feed flow velocities, although the differences are not significant. 

Using different CP correlations (Case 6 to 9), the largest differences are −4.7% in recovery and 3.4% in pressure drop (Case 8 at Condition 1). Results obtained with our new correlation for the zigzag configuration has the smallest relative difference in recovery up to a feed velocity of 0.2 m/s. At higher flow velocities, the correlation for the submerged configuration appears to have the smallest relative difference.

Although using different mass transfer and pressure drop correlations do not appear to have a strong influence on the predicted performance of a single pressure vessel, the effect could be more significant in a larger system that uses multiple pressure vessels in parallel. Accurate predictions are even more important for process design when the number of membrane modules and pressure vessels in a plant are determined based on a predictive model. For example, if a single stage RO plant with a capacity of 3 million/day is to be built, using the correlations adopted in Case 1 would predict the required number of pressure vessels to be approximately 34,705 whereas those at for Case 8 would predict 36,413. This discrepancy will lead to different estimates of capital and maintenance cost. Therefore, the selection of correlations implemented in an overall process model is important.

### 3.5. Limitations of the CFD Model and Derived Correlations

Although the CFD model adopted in this study accounts for the presence of spacers and membrane permeation and the simulations cover a wide range of operating and geometric conditions, the present study has two main limitations. Firstly, the boundary condition for permeable walls implemented in the CFD model needs to be experimentally validated, although the solution–diffusion model is widely accepted for RO membranes. Owing to the lack of experimental data obtained in the presence of spacers and RO membranes, indirect comparisons of CP moduli are made with existing correlations in the literature. Secondly, the spacer-filled fluid domain is simplified to allow for a large number of simulations. In particular, 2D representations of spacer-filled channels do not account for mesh and flow attack angles. The degree of flow recirculation and stagnation in 2D geometries might differ from that in 3D geometries when angles are varied, which would affect CP and pressure drop. Thus, it is necessary to compare 2D and 3D CFD models with various spacer geometries in order to determine whether 2D approximations are sufficient for the purpose of performance and energy prediction.

## 4. Conclusions

CFD models for spacer-filled feed channels in an RO membrane module are built for three commonly adopted configurations. By incorporating the solution–diffusion model, permeation phenomena through RO membranes can be captured in CFD simulations, allowing us to predict membrane performance under a wide range of operating and geometric conditions. Using results obtained from 228 CFD simulations, new correlations for CP modulus and friction factor are obtained, which fit the CFD data very well with a maximum absolute percentage error of 1.82% for CP modulus and 6.13% for pressure drop. A distinct advantage of our correlations for CP modulus is that they can direct predict the impact of permeate flux on the polarized concentration at membrane walls, bypassing the additional steps needed when using existing correlations based on Sherwood number.

Comparisons of our new correlations for CP modulus and the selected mass transfer correlations show that predicted water fluxes are comparable with a mean difference of less than 7%. The correlations for pressure drop, on the other hand, have a larger discrepancy, up to 84%. When implemented in a process model, the predicted performance and energy consumption for a single pressure vessel with six membrane modules differ by 4.7% for recovery and 1.6% for specific energy consumption. It is, therefore, concluded that this approach to predict a process-level system by exploiting new correlations derived from CFD results demonstrates its versatile applicability to various types of spacers and modules unlike the membrane manufacturer’s projection software, which can only predict their own products.

## Figures and Tables

**Table 1:** Model parameters and simulation conditions where inlet concentration *c*_0_, solute diffusivity in water *D_sw_*, outlet pressure *p*_0_, inlet velocity *u*_0_, channel height *H_c_*, and filament spacing to channel height ratio *L_f_*-to-*H_c_* are varied.

Parameters and Conditions	Case Number					
	Base	1–7	8–15	16–19	20–30	31–75
Hydraulic conductivity, *A* (m/(s·Pa))	2.50 × 10^−12^					
Solute permeability, *B* (m/s)	2.50 × 10^−8^					
Osmotic factor, *κ* (m^3^·Pa/mol)	4958					
Density, *ρ* (kg/m^3^)	998.20					
Viscosity, *μ* (Pa·s)	8.93 × 10^−4^					
Inlet concentration, *c*_0_ (mol/m^3^)	603.45	362.07–905.17	603.45	603.45	603.45	603.45
Solute diffusivity, *D_sw_* (10^−9^ m^2^/s)	1	1	0.2–8	1	1	1
Outlet pressure, *p*_0_ (10^5^ Pa)	60	60	60	50–90	60	60
Mean inlet velocity, *u*_0_ (m/s)	0.1	0.1	0.1	0.1	0.01–0.35	0.1
Channel height, *H_c_* (10^−3^ m)	1	1	1	1	1	0.2–1.4
Filament spacing to channel height ratio, *L_f_*/*H_c_* (-)	4.5	4.5	4.5	4.5	4.5	2–6

**Table 2:** List of parameters for RO process simulation.

Symbol (Unit)	Name	Value	Source
*N_ele_* (-)	Number of modules arrayed in series	6	[49,50,51]
*N_leaf_* (-)	Number of leaves	16	[42,52]
*L_i_* (m)	Membrane length	1.016	[52]
*W_i_* (m)	Membrane width	1.505	[42,52]
*H_c_* (m)	Feed spacer thickness	7.1 × 10^−4^	[52]
*H_c_*_,*p*_ (m)	Permeate spacer thickness	4.0 × 10^−4^	[52]
*L_g_* (m)	Glue line width in length direction	4 × 10^−2^	[53]
*W_g_* (m)	Glue line width in width direction	17 × 10^−2^	[53]
*D_f_* (m)	Filament diameter	3.85 × 10^−4^	[30]
*L_f_* (m)	Filament spacing	2.8 × 10^−3^	[30]
*ε* (-)	Voidage of spacer-filled channel	0.89	[30]
*d_h_* (m)	Hydraulic diameter of spacer-filled channel	0.95 × 10^−3^	[30]
*A* (m^3^/m^2^ s Pa)	Pure water permeability constant	3.26 × 10^−12^	Estimated
*B* (m^3^/m^2^ s)	Salt permeability constant	1.15 × 10^−8^	Estimated
*k_sp_*_,*f*_ (-)	Frictional coefficient in feed channel	1.58	Estimated
*k_sp_*_,*p*_ (-)	Frictional coefficient in permeate channel	30	Estimated
*η_HPP_* (-)	Efficiency of high pressure pump	0.85	[42,49,50,51]
*η_PX_* (-)	Efficiency of pressure exchanger	0.98	[42,49,50,51]

**Table 3:** Estimated parameter values for CP modulus and pressure drop correlations in three configurations and APEs of the correlations compared to the corresponding CFD results.

		Regime	Cavity		Submerged	Zigzag	
			*Re_c_* ≥ *Re_crit_*	*Re_c_* < *Re_crit_*		*Re_c_* ≥ *Re_crit_*	*Re_c_* < *Re_crit_*
CP modulus, *M_CP_* in Equation (16)	Estimated parameter values	*C*	2.55 × 10^−4^	7.06 × 10^−4^	5.55 × 10^−3^	3.24 × 10^−4^	7.63 × 10^−4^
		*α*	−0.350	−0.382	−0.422	−0.394	−0.365
		*β*	1.11	0.931	1.09	1.12	0.909
		*γ*	0.611	0.611	0.672	0.597	0.597
		*δ*	5.40 × 10^−4^	−0.143	0.536	2.18 × 10^−4^	−0.112
	Mean APE [%]		0.375		0.147	0.516	
Friction factor, *f* in Equation (17)	Estimated parameter values	*C*′	14.8		63.3	14.0	
		*α*′	−0.910		−0.994	−0.898	
		*δ*′	−0.525		−0.810	−0.510	
		*ζ*	0.0256		0.0868	0.0200	
	Mean APE [%]		1.15		1.48	0.974	

**Table 4:** Average and maximum absolute percentage difference between the new correlations and existing correlations for CP moduli and water fluxes.

	Correlation	Absolute Percentage Difference, |*Y* – *Y_ex_*| /*Y_ex_* (%)					
Variable	Existing	Schock and Miquel [30]			Koutsou et al. [27]		
	New	Cavity	Submerged	Zigzag	Cavity	Submerged	Zigzag
CP modulus	Mean	1.9	4.5	1.8	5.5	1.8	5.4
	Max.	6.7	10.3	6.5	5.6	1.9	5.6
Water flux	Mean	2.7	6.4	2.6	5.6	1.6	5.5
	Max.	11.0	16.5	10.7	5.8	1.7	6.1

**Table 5:** Average and maximum absolute percentage differences between our new correlations and existing correlations for pressure gradient.

Correlation	Absolute Percentage Difference (%)								
Existing	Schock and Miquel [30]			Koutsou et al. [26] (*L_f_*/*D_f_* = 8)			Koutsou et al. [26] (*L_f_*/*D_f_* = 6)		
New	Cavity	Submerged	Zigzag	Cavity	Submerged	Zigzag	Cavity	Submerged	Zigzag
Mean	54	12	55	66	30	67	80	58	80
Max.	63	28	64	74	44	75	84	64	84

**Table 6:** Simulation results for water recovery *R_w_*, specific energy consumption *E_sp_* and feed pressure drop *P_d_*, with different combinations of CP and pressure drop correlations under three simulation conditions (with feed concentration *c_f_* = 35kg/m^3^).

Case No.	Correlation		Condition 1			Condition 2			Condition 3		
	CP	d*p*/d*x*	*Q_f_* = 2 × 10^−3^ m^3^/s (*u_f_* = 0.13 m/s), *P_f_* = 60 bar			*Q_f_* = 3 × 10^−3^ m^3^/s (*u_f_* = 0.20 m/s), *P_f_* = 70 bar			*Q_f_* = 4.5 × 10^−3^ m^3^/s (*u_f_* = 0.30 m/s), *P_f_* = 80 bar		
			*R_w_* (%)	*E_sp_* (kWh/m^3^)	*P_d_* (×10^5^ Pa)	*R_w_* (%)	*E_sp_* (kWh/m^3^)	*P_d_* (×10^5^ Pa)	*R_w_* (%)	*E_sp_* (kWh/m^3^)	*P_d_* (×10^5^ Pa)
1	Equation (25)	Equation (26)	50.03	2.06	0.72	53.43	2.39	1.08	52.92	2.74	1.67
Relative difference [%]			Condition 1			Condition 2			Condition 3		
			Δ*_Rw_*	Δ*_Esp_*	Δ*_Pd_*	Δ*_Rw_*	Δ*_Esp_*	Δ*_Pd_*	Δ*_Rw_*	Δ*_Esp_*	Δ*_Pd_*
2	Equation (25)	Zig *	0.53	−0.64	−53	0.56	−0.66	−49	0.65	−0.81	−44
3	Equation (25)	Sub	0.066	−0.096	−8.0	−0.056	0.034	2.3	−0.36	0.37	19
4	Equation (25)	S&M ^+^	0.31	−0.40	−34	0.075	−0.15	−12	−0.43	0.39	20
5	Equation (25)	KT8 ^$^	0.031	−0.097	−8.4	−0.42	0.39	28	−1.5	1.6	83
6	Zig *	Equation (26)	0.12	−0.0076	0.34	−1.0	0.10	1.1	−2.6	0.28	1.6
7	Sub	Equation (26)	1.6	−0.15	−1.0	1.1	−0.10	-0.49	0.25	−0.023	0.081
8	S&M ^++^	Equation (26)	-4.7	0.49	3.4	−4.7	0.47	2.9	−4.3	0.47	2.1
9	KT ^$$^	Equation (26)	1.9	−0.18	−1.5	1.9	−0.18	−1.2	1.7	−0.17	−0.78

* The correlation for the zigzag configuration is chosen due to negligible difference between the cavity and zigzag configurations. ^+^ The correlation of Schock and Miquel for pressure gradient [30] in Equation (23). ^++^ The correlation of Schock and Miquel [30] for mass transfer coefficient in Equation (20). ^$^ The correlation of Koutsou et al. [26] for pressure gradient when *L_f_*/*D_f_* = 8 in Equation (24); ^$$^ The correlation of Koutsou et al. [27] for mass transfer coefficient in Equation (21).

## Data Availability

The data presented in this study are available upon request from the corresponding author.

## Supplementary Materials

The following are available online at https://www.mdpi.com/article/10.3390/membranes11050338/s1, Section A: Derivation of correlations: dimensional analysis, Section B: B. Supplementary CFD simulation results, Section C: C. Data fitting to estimate parameters in derived correlations.
