# Supplementary material for: Correlations for Concentration Polarization and Pressure Drop in Spacer-Filled RO Membrane Modules Based on CFD Simulations
Source: Membranes (Basel). 2021 May 1;11(5):338. doi: 10.3390/membranes11050338 (PMC8147287; doi:10.3390/membranes11050338)
Supplement: Supplementary file 1 [file membranes-11-00338-s001.zip › membranes-1199846-supplementary.pdf]

membranes

Supplementary Materials: Correlations for Concentration Polarization and Pressure Drop in Spacer-Filled RO Membrane Modules Based on CFD Simulations

Boram Gu [1,2,*], Claire S. Adjiman [1] and Xiao Yun Xu [1,*]

[1] Department of Chemical Engineering, Imperial College London, United Kingdom; c.adjiman@imperial.ac.uk
[2] School of Chemical Engineering, Chonnam National University, Republic of Korea
* Correspondence: b.gu13@imperial.ac.uk (B.G.); yun.xu@imperial.ac.uk (X.Y.X); Tel.: +44-20-7594-5588 (X.Y.X)

1. Introduction

A. Derivation of correlations: dimensional analysis

A.1 Correlation for CP modulus

A total of 8 variables, which are considered to affect mass transfer, are selected: CP modulus M_{CP}, fluid density ρ, dynamic viscosity μ, solute diffusivity in fluid D_{sw}, cross-velocity u_c, transmembrane velocity u_t (or water flux at membrane walls J_w), filament diameter D_f and filament spacing L_f. All the variables consist of three dimensions, length [L], mass [M] and time [t]. According to the Buckingham's π theorem, the number of dimensionless π-variables is determined to be 5, as a result of subtracting the number of dimensions from the number of variables (8 − 3 = 5). To construct π-variables, the fluid density ρ, dynamic viscosity μ, filament diameter D_f are chosen to be a repeating variables. As a result, five π-variables are formed as follows:

$$\pi_1 = \rho^{a_1}\mu^{b_1}D_f^{\,c_1}D_{sw} = \left[\frac{M}{L^3}\right]^{a_1}\left[\frac{M}{Lt}\right]^{b_1}\left[L\right]^{c_1}\left[\frac{L^2}{t}\right] \quad \text{(A.1)}$$

$$\pi_2 = \rho^{a_2}\mu^{b_2}D_f^{\,c_2}u_c = \left[\frac{M}{L^3}\right]^{a_2}\left[\frac{M}{Lt}\right]^{b_2}\left[L\right]^{c_2}\left[\frac{L}{t}\right] \quad \text{(A.2)}$$

$$\pi_3 = \rho^{a_3}\mu^{b_3}D_f^{\,c_3}u_t = \left[\frac{M}{L^3}\right]^{a_3}\left[\frac{M}{Lt}\right]^{b_3}\left[L\right]^{c_3}\left[\frac{L}{t}\right] \quad \text{(A.3)}$$

$$\pi_4 = \rho^{a_4}\mu^{b_4}D_f^{\,c_4}L_f = \left[\frac{M}{L^3}\right]^{a_4}\left[\frac{M}{Lt}\right]^{b_4}\left[L\right]^{c_4}\left[L\right] \quad \text{(A.4)} \quad \text{(A.4)}$$

$$\pi_5 = \rho^{a_5}\mu^{b_5}D_f^{\,c_5}M_{CP} = \left[\frac{M}{L^3}\right]^{a_5}\left[\frac{M}{Lt}\right]^{b_5}\left[L\right]^{c_5}\left[1\right] \quad \text{(A.5)}$$

The exponents in Equations (A.1) to (A.5) can be found via dimensional analysis. For example, in Equation (A.1), by comparing exponents on the left and right hand sides, three equations are established for three dimensions, [M], [L] and [t].

For [M], $a_1 + b_1 = 0$

For [L], $-3a_1 - b_1 + c_1 + 2 = 0$

For [t], $-b_1 - 1 = 0$

By solving three simultaneous equations, a_1, b_1 and c_1 are obtained to be 1, –1 and 0, respectively. As a result, π-variables are:

$$\pi_1 = \rho^1 \mu^{-1} D_f^{\ 0} D_{sw} = \frac{\rho D_{sw}}{\mu} \tag{A.6}$$

$$\pi_2 = \rho^1 \mu^{-1} D_f^{\ 1} u_c = \frac{\rho u_c D_f}{\mu} \tag{A.7}$$

$$\pi_3 = \rho^1 \mu^{-1} D_f^{\ 1} u_t = \frac{\rho u_t D_f}{\mu} \tag{A.8}$$

$$\pi_4 = \rho^0 \mu^0 D_f^{\ -1} L_f = \frac{L_f}{D_f} \tag{A.9}$$

$$\pi_5 = \rho^0 \mu^0 D_f^{\ 0} M_{CP} = M_{CP} \tag{A.10}$$

The fifth π-variable, π_5 is identical to the CP modulus. The inverse of π_1 is Schmidt number and π_2 and π_3 are of a commonly used dimensionless number, Reynold number. Both are widely used to describe mass transfer. One π-variable can be expressed as a function of other π-variables. Since we aim to derive a correlation for CP, π_5 is expressed as a function of (π_1, π_2, π_3, π_4). These π-variables can be converted to more physically meaningful numbers as presented in Equations (12) to (15) in the manuscript.

A.2 Correlation for pressure drop

For a pressure drop, 6 variables are selected to comprise a correlation: pressure gradient dp/dx, cross-velocity u_c, fluid density ρ, dynamic viscosity μ, filament diameter D_f and filament spacing L_f. These variables contain three dimensions, length [L], mass [M] and time [t]. As a result, there are three π-variables (6 − 3 = 3). The same procedure as described above applies here.

$$\pi_1 = \rho^{a_1} u_c^{\ b_1} D_f^{\ c_1} \frac{dp}{dx} = \left[\frac{M}{L^3}\right]^{a_1} \left[\frac{L}{t}\right]^{b_1} [L]^{c_1} \left[\frac{M}{L^2 t^2}\right] \rightarrow \pi_1 = \rho^{-1} u_c^{\ -2} D_f^{\ 1} \frac{dp}{dx} = \frac{D_f}{\rho u_c^{\ 2}} \frac{dp}{dx} \tag{A.11}$$

$$\pi_2 = \rho^{a_2} u_c^{\ b_2} D_f^{\ c_2} L_f = \left[\frac{M}{L^3}\right]^{a_2} \left[\frac{L}{t}\right]^{b_2} [L]^{c_2} [L] \rightarrow \pi_2 = \rho^0 u_c^{\ 0} D_f^{\ -1} L_f = \frac{L_f}{D_f} \tag{A.12}$$

$$\pi_3 = \rho^{a_3} u_c^{\ b_3} D_f^{\ c_3} \mu = \left[\frac{M}{L^3}\right]^{a_3} \left[\frac{L}{t}\right]^{b_3} [L]^{c_3} \left[\frac{M}{Lt}\right] \rightarrow \pi_3 = \rho^{-1} u_c^{\ -1} D_f^{\ -1} \mu = \frac{\mu}{\rho u_c D_f} \tag{A.13}$$

The first π-variable, π_1, is defined as a friction factor, f, and the rest are already defined in Equations (A.7) and (A.9).

B. Supplementary CFD simulation results

Some of velocity vectors and the profiles of concentration at the lower and upper membrane walls are presented in this section in order to provide an additional explanation for the analysis presented in the manuscript.

Velocity vectors and concentration profiles at membrane walls in the cavity configuration under selected conditions are presented in Figures S1 and S2. Recirculation zones are observed in the proximity of spacer filaments. As a flow velocity increases, the recirculation zones become extended in the cavity configuration. Similar behaviours can be seen in the zigzag configuration in Figure S8. For the submerged configuration shown in Figures S5 and S7, stagnation zones ahead of spacer filaments are created in the middle of channels and their sizes vary depending on the flow velocity and geometric conditions.

Concentration profiles are also displayed for the lower and upper membrane walls with respect to the normalised distance in Figure S2. Peaks appear in the cavity configuration, the locations of which correspond to those of filaments in contact with membrane walls. They reach almost up to 1,500 mol/m^3, which is more than twice the inlet concentration. On the other hand, the submerged configuration does not exhibit peaks in the concentration profiles as shown in Figure S6 and concentration hardly exceeds 900 mol/m^3. An interesting finding is that the width of peaks varies with an inlet velocity in the cavity configuration; increasing an inlet velocity leads to a narrower peak. This can be explained in connection with the sizes of flow recirculation zones. In addition to recirculation zones there exist stagnation zones adjacent to spacer filaments, where a high concentration build-up occurs. At high velocities, the convective mixing is enhanced, thereby reducing the size of stagnation zones.

The influences of geometric variables can also be investigated using Figures S3, S7 and S9, where velocity vectors for the cavity, submerged and zigzag configurations with different channel heights and two L_f-to-D_f ratios are displayed, respectively. A general trend is that recirculation zones become broader with an increase in a channel height at a fixed L_f-to-D_f ratio; accordingly concentration peaks become sharper, as shown in Figure S4 for the cavity configuration. In comparison between different L_f-to-D_f ratios at same channel heights, concentration peaks at an L_f-to-D_f ratio of 10 are sharper than those at an L_f-to-D_f ratio of 4. It can be noticed that there exist two distinct hills at L_f-to-D_f = 4 in Figure S4 (b) in the middle of the channel while two hills are attached very closely in geometries with higher L_f-to-D_f ratios. This can be explained by two recirculation zones formed between filaments, as can be observed in Figure S3 (b).

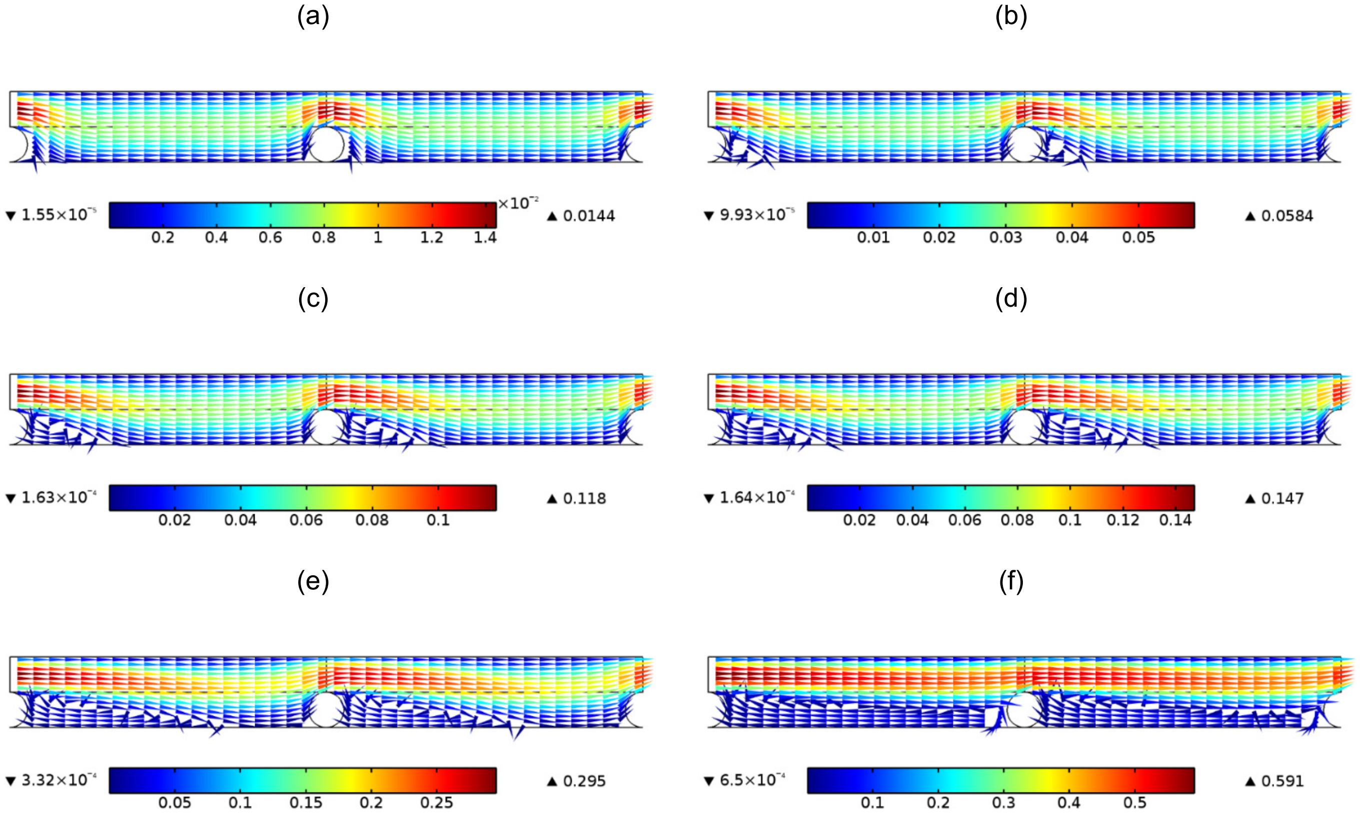

Figure S1. Velocity vectors in the cavity configuration under different inlet velocities. (a) u_0 = 0.01 m/s, (b) u_0 = 0.04 m/s, (c) u_0 = 0.08 m/s, (d) u_0 = 0.1 m/s, (e) u_0 = 0.2 m/s and (f) u_0 = 0.35 m/s.

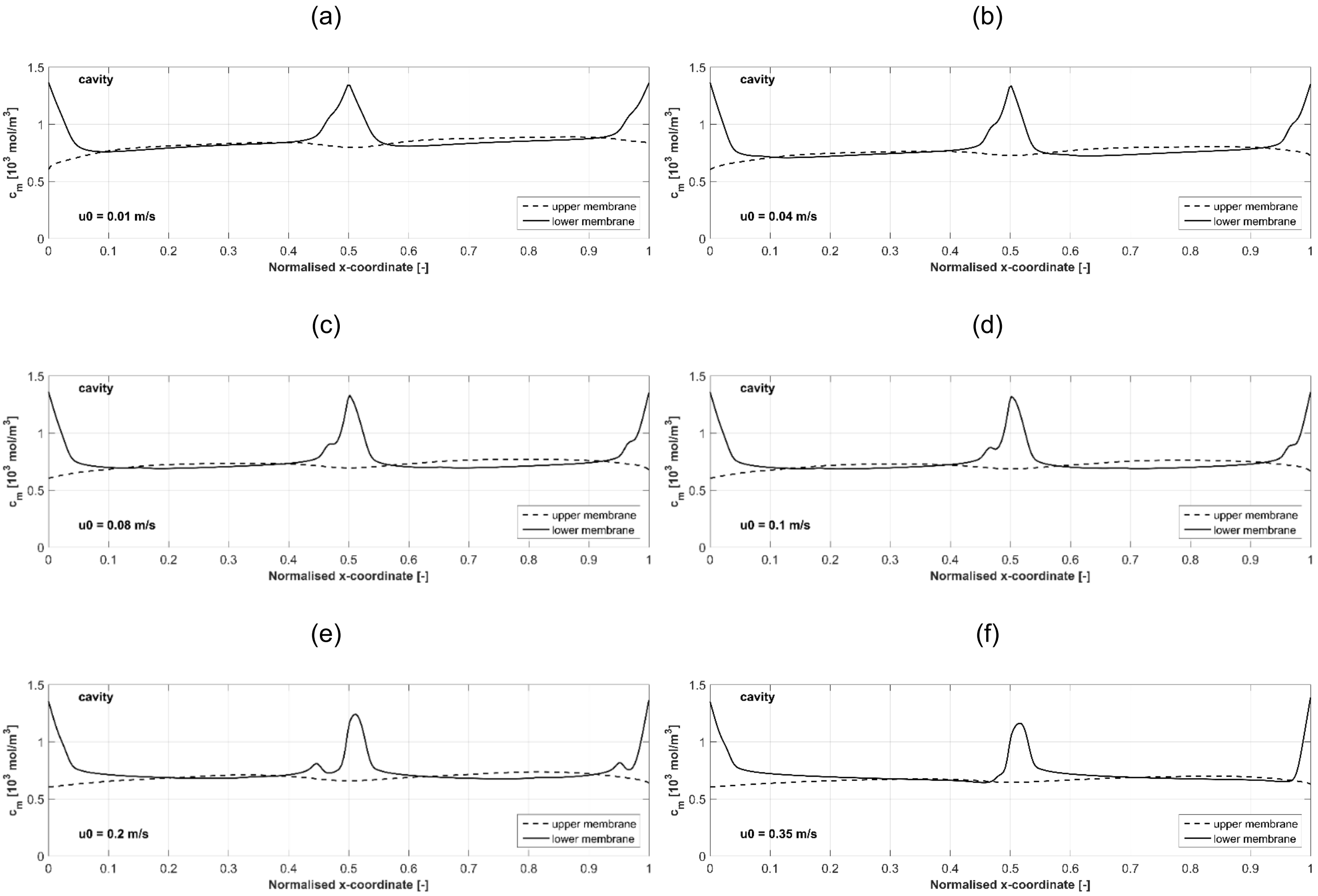

Figure S2. Local concentration at upper and lower membrane walls in the cavity configuration under varying inlet velocity. (a) u_0 = 0.01 m/s, (b) u_0 = 0.04 m/s, (c) u_0 = 0.08 m/s, (d) u_0 = 0.1 m/s, (e) u_0 = 0.2 m/s and (f) u_0 = 0.35 m/s

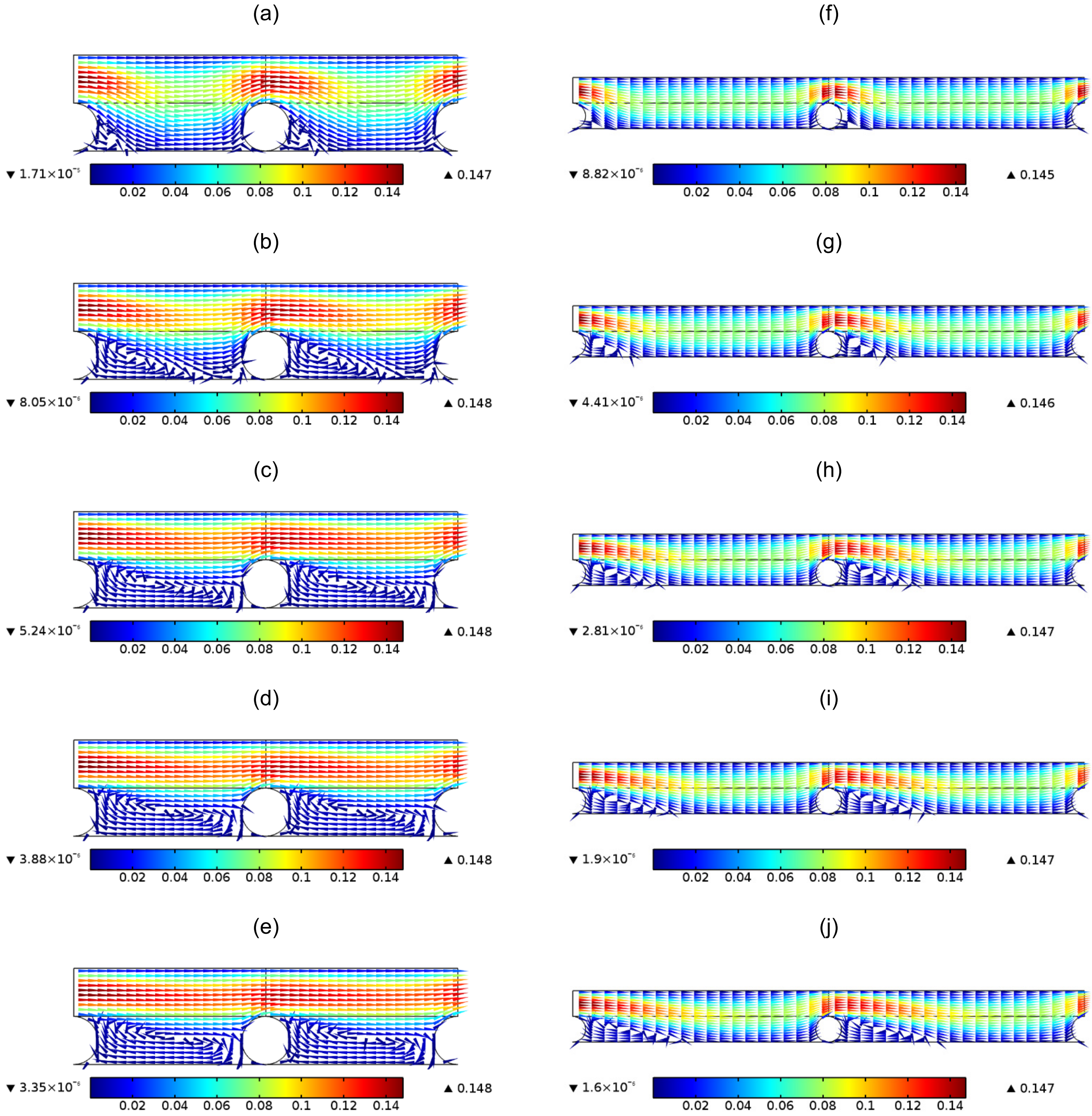

Figure S3. Velocity vectors in the cavity configuration under different channel. (a) L_f-to-H_c ratio = 2 and H_c = 0.2 mm, (b) L_f-to-H_c ratio = 2 and H_c = 0.6 mm, (c) L_f-to-H_c ratio = 2 and H_c = 1.0 mm, (d) L_f-to-H_c ratio = 2 and H_c = 1.2 mm, (e) L_f-to-H_c ratio = 2 and H_c = 1.4 mm, (f) L_f-to-H_c ratio = 5 and H_c = 0.2 mm, (g) L_f-to-H_c ratio = 5 and H_c = 0.6 mm, (h) L_f-to-H_c ratio = 5 and H_c = 1.0 mm, (i) L_f-to-H_c ratio = 5 and H_c = 1.2 mm and (j) L_f-to-H_c ratio = 5 and H_c = 1.4 mm.

(a)

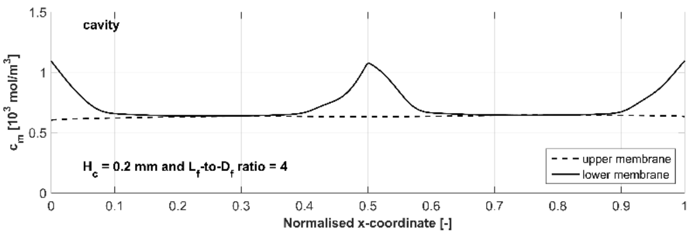

(b)

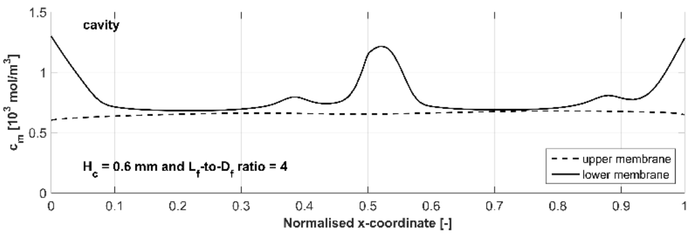

(c)

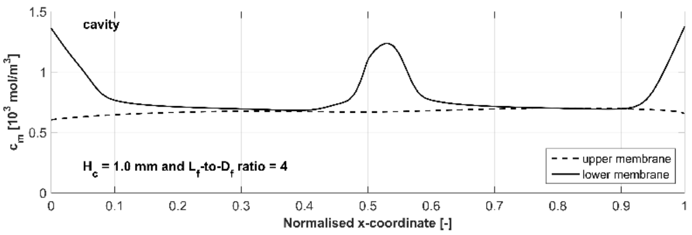

(d)

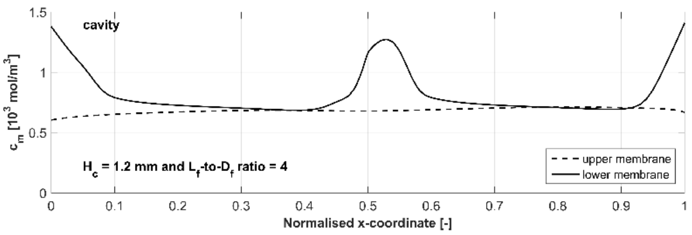

(e)

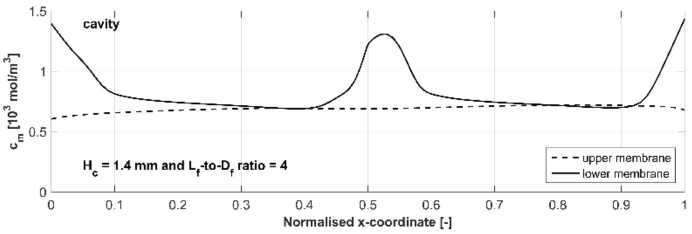

(f)

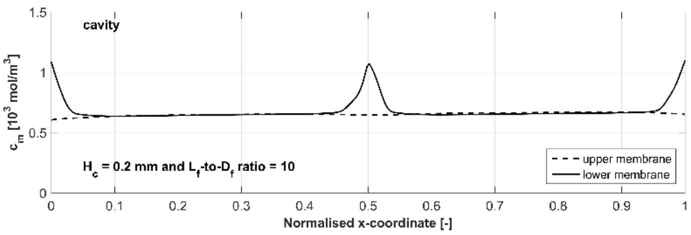

(g)

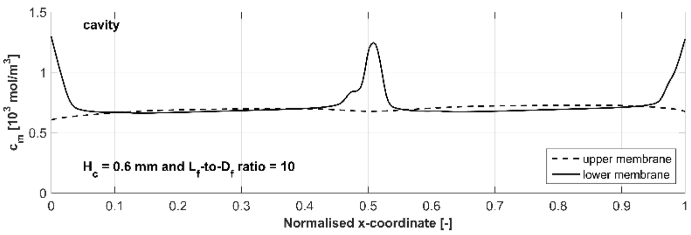

(h)

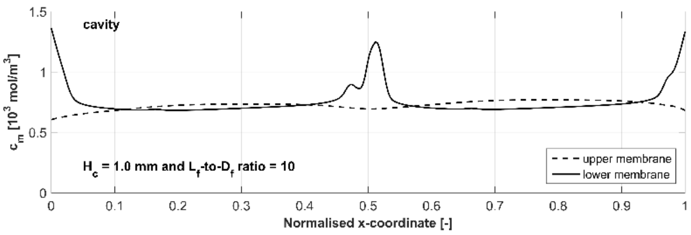

(i)

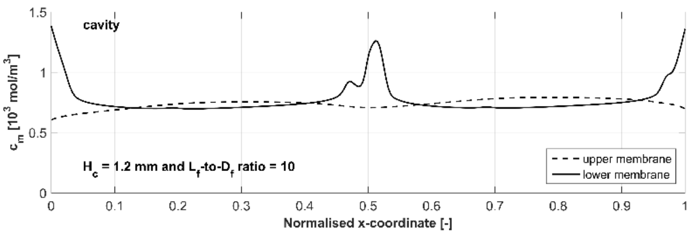

(j)

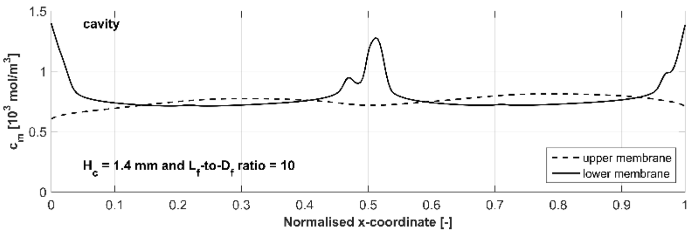

Figure S4. Local concentration at upper and lower membrane walls in the cavity configuration. (a) L_f-to-H_c ratio = 2 and H_c = 0.2 mm, (b) L_f-to-H_c ratio = 2 and H_c = 0.6 mm, (c) L_f-to-H_c ratio = 2 and H_c = 1.0 mm, (d) L_f-to-H_c ratio = 2 and H_c = 1.2 mm, (e) L_f-to-H_c ratio = 2 and H_c = 1.4 mm, (f) L_f-to-H_c ratio = 5 and H_c = 0.2 mm, (g) L_f-to-H_c ratio = 5 and H_c = 0.6 mm, (h) L_f-to-H_c ratio = 5 and H_c = 1.0 mm, (i) L_f-to-H_c ratio = 5 and H_c = 1.2 mm and (j) L_f-to-H_c ratio = 5 and H_c = 1.4 mm.

(a) (b)

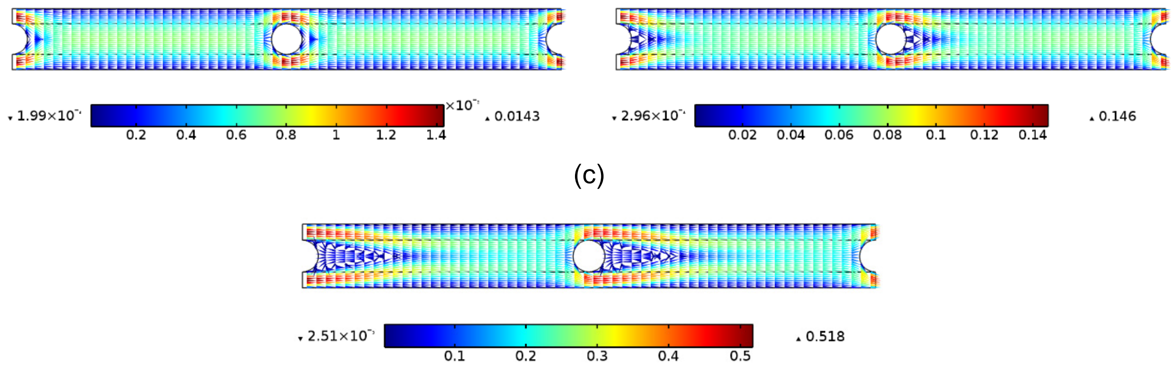

Figure S5. Velocity vectors in the submerged configuration under different inlet velocities. (a) u_0 = 0.01 m/s, (b) u_0 = 0.1 m/s, (c) u_0 = 0.35 m/s.

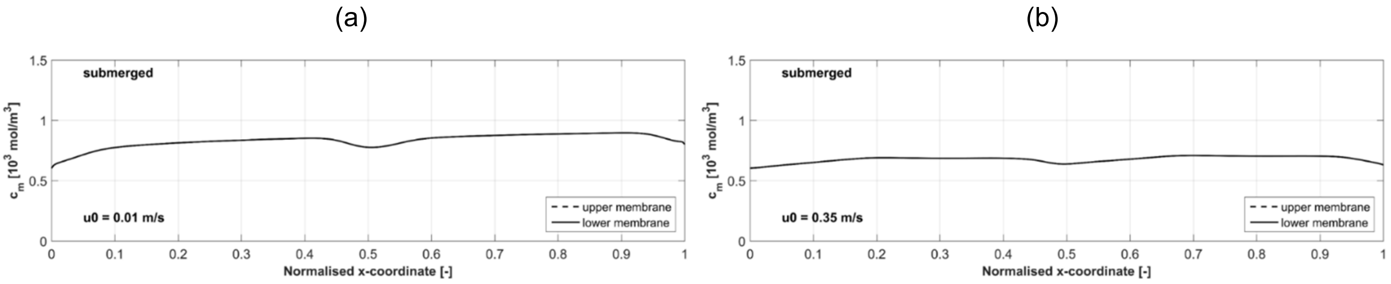

Figure S6. Local concentration at upper and lower membrane walls in the submerged configuration under varying inlet velocity. (a) u_0 = 0.01 m/s and (b) u_0 = 0.35 m/s.

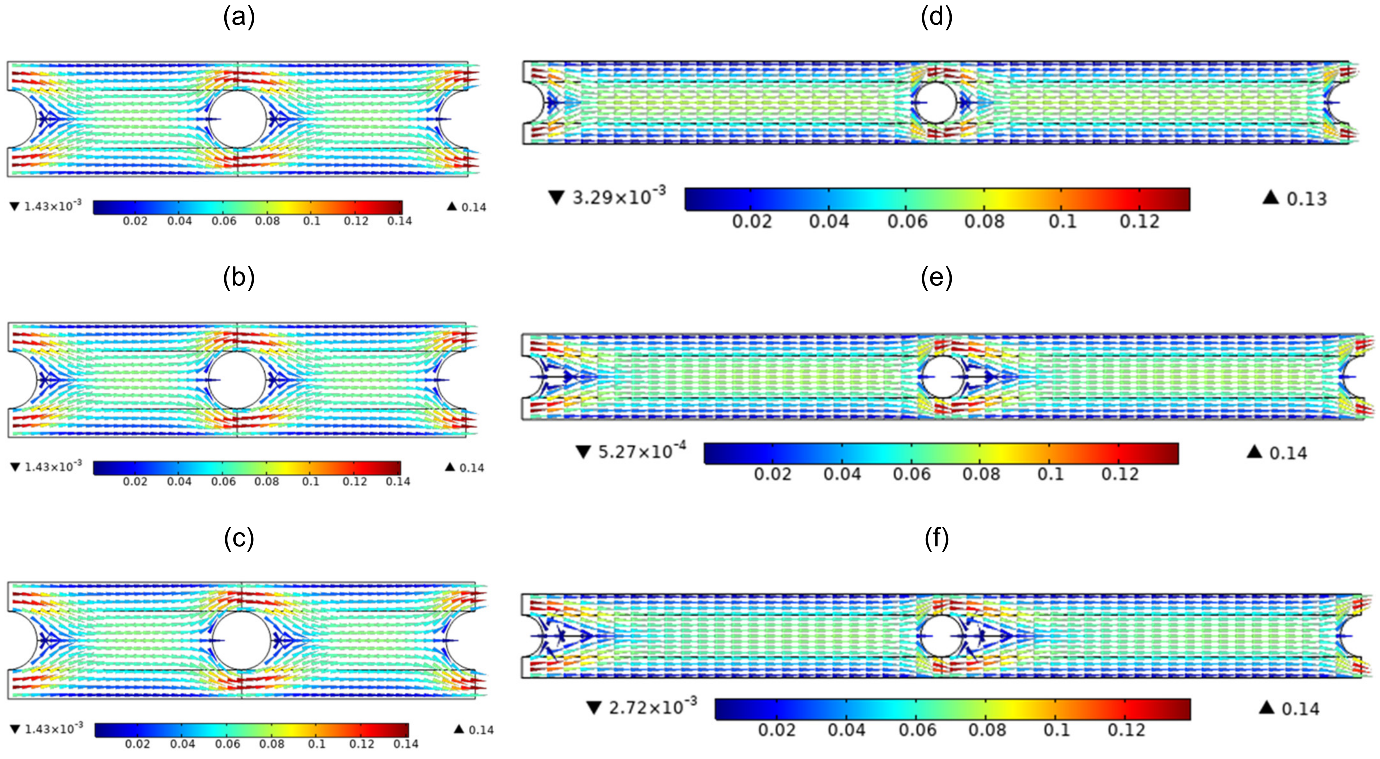

Figure S7. Velocity vectors in the submerged configuration under different channel. (a) L_f-to-H_c ratio = 2, H_c = 0.2 mm, (b) L_f-to-H_c ratio = 2 and H_c = 1.0 mm, (c) L_f-to-H_c ratio = 2 and H_c = 1.4 mm, (d) L_f-to-H_c ratio = 5 and H_c = 0.2 mm, (e) L_f-to-H_c ratio = 5 and H_c = 1.0 mm, (f) L_f-to-H_c ratio = 5 and H_c = 1.4 mm.

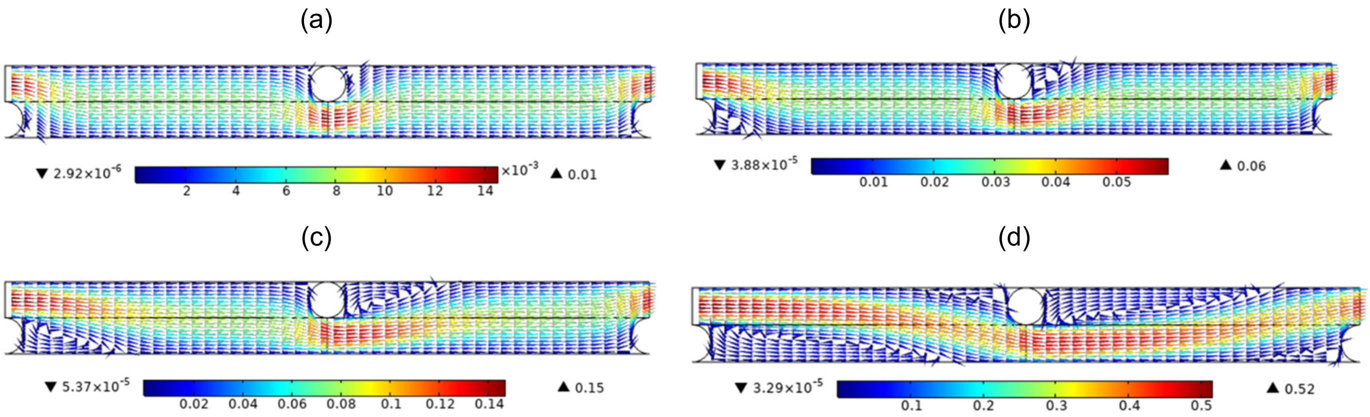

Figure S8. Velocity vectors in the zigzag configuration under different inlet velocities. (a) $u_0 = 0.01$ m/s, (b) $u_0 = 0.04$ m/s, (c) $u_0 = 0.1$ m/s, (d) $u_0 = 0.35$ m/s.

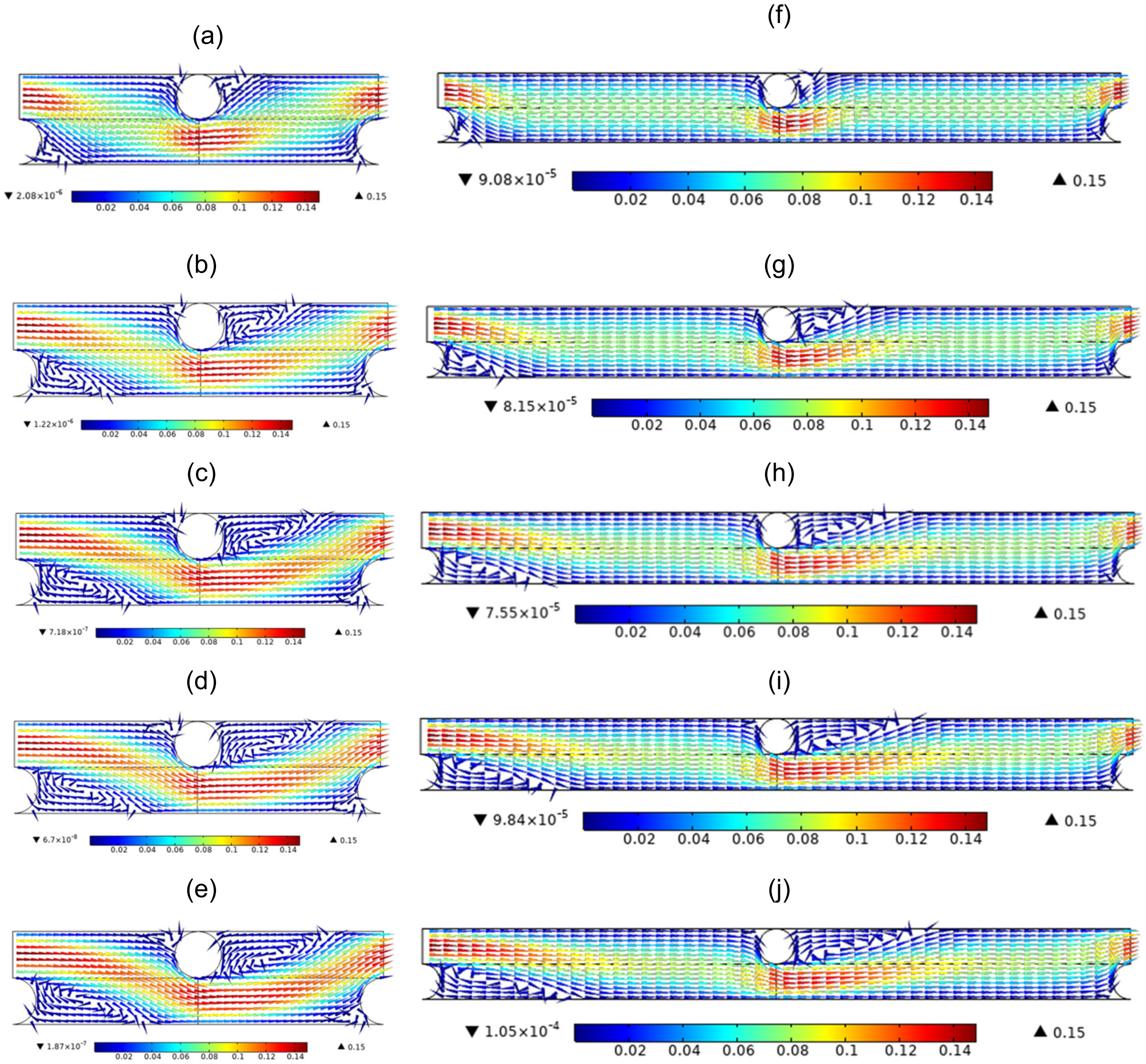

Figure S9. Velocity vectors in the zigzag configuration under different channel. (a) L_f-to-H_c ratio = 2 and $H_c = 0.2$ mm, (b) L_f-to-H_c ratio = 2 and $H_c = 0.6$ mm, (c) L_f-to-H_c ratio = 2 and $H_c = 1.0$ mm, (d) L_f-to-H_c ratio = 2 and $H_c = 1.2$ mm, (e) L_f-to-H_c ratio = 2 and $H_c = 1.4$ mm, (f) L_f-to-H_c ratio = 5 and $H_c = 0.2$ mm, (g) L_f-to-H_c ratio = 5 and $H_c = 0.6$ mm, (h) L_f-to-H_c ratio = 5 and $H_c = 1.0$ mm, (i) L_f-to-H_c ratio = 5 and $H_c = 1.2$ mm and (j) L_f-to-H_c ratio = 5 and $H_c = 1.4$ mm.

C. Data fitting to estimate parameters in derived correlations

A graphical representation of data fitting and relative errors for the cavity configuration is also available here, as a representative case.

Concentration polarisation modulus, M_{CP}

In Figures S10 and S11, upper graphs present CP moduli from CFD simulations (blue squares) and calculations by the derived correlation (orange triangles) and lower bar graphs show relative errors between the CFD results and correlations. It is seen that relative errors are below 2 %.

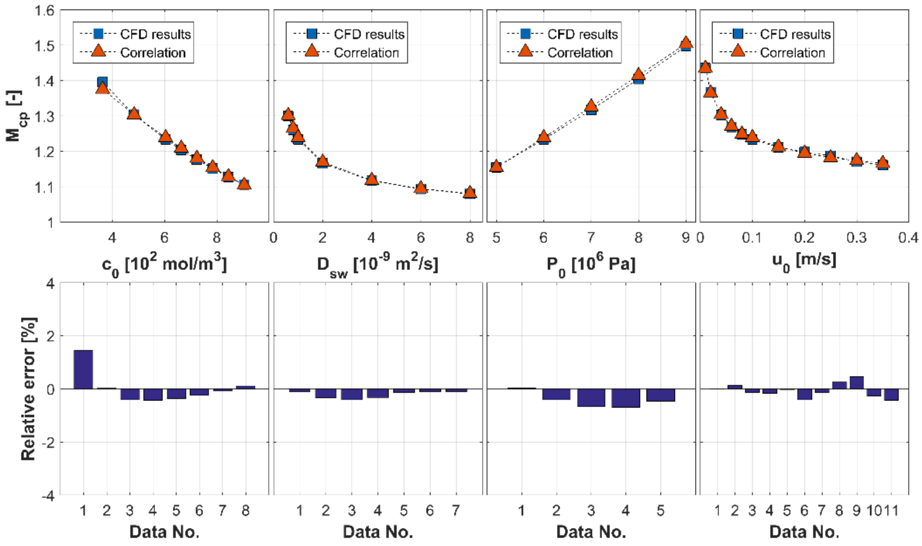

Figure S10. Comparison of CFD results and calculations by the derived correlation under varying operating conditions for the cavity configuration.

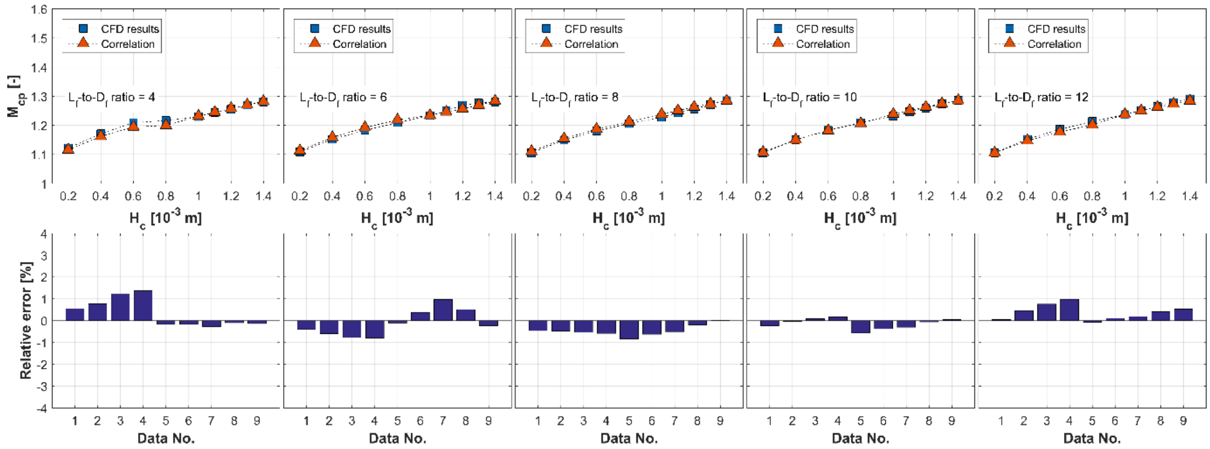

Figure S11. Comparison of CFD results and calculations by the derived correlation under varying geometric conditions for the cavity configuration.

Friction factor, f

The upper graphs display the value of friction factor calculated using CFD results and Equation (18). Triangular and square symbols denote CFD results and correlations, respectively. Although relative errors for the friction correlation are larger than those for the CP correlation, they are still below 5 %.

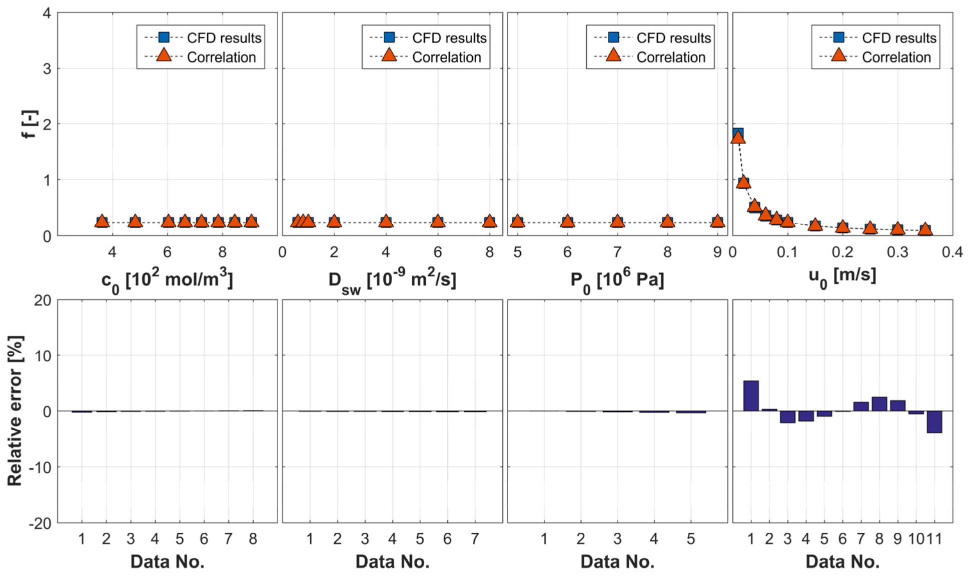

Figure S12. Comparison of CFD results and calculations by the derived correlation under varying operating conditions for the cavity configuration.

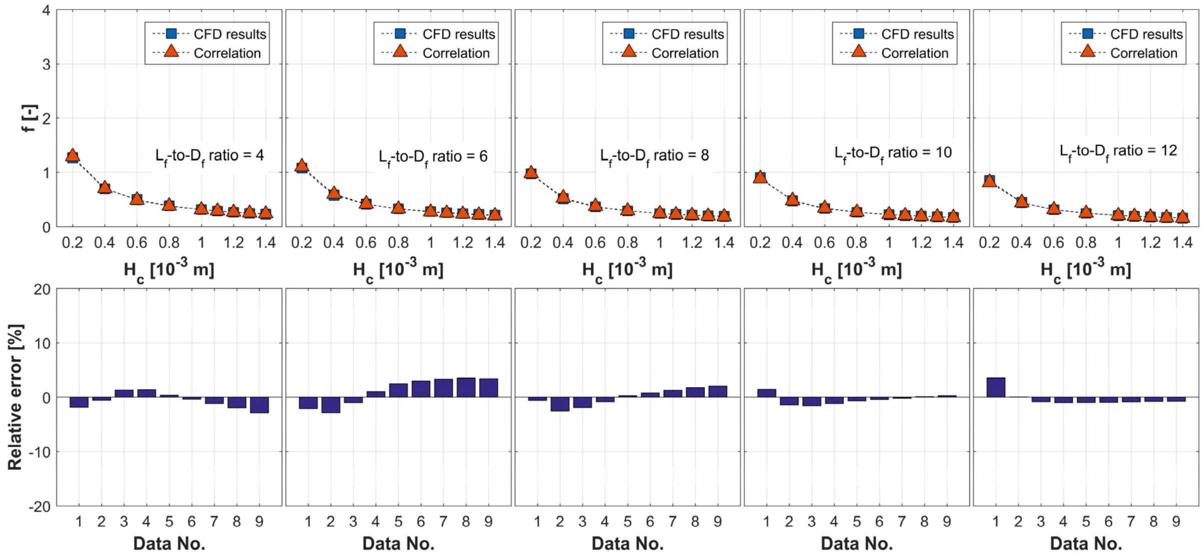

Figure S13. Comparison of CFD results and calculations by the derived correlation under varying geometric conditions for the cavity configuration.
